# Critical role of quorum sensing-dependent glutamate metabolism in homeostatic osmolality and outer membrane vesiculation in *Burkholderia glumae*

**DOI:** 10.1038/srep44195

**Published:** 2017-03-08

**Authors:** Yongsung Kang, Eunhye Goo, Jinwoo Kim, Ingyu Hwang

**Affiliations:** 1 Department of Agricultural Biotechnology, Seoul National University, Seoul 08826, Republic of Korea; 2 Division of Applied Life Science and Institute of Agriculture and Life Sciences, Gyeongsang National University, Jinju 52828, Republic of Korea

## Abstract

Metabolic homeostasis in cooperative bacteria is achieved by modulating primary metabolism in a quorum sensing (QS)-dependent manner. A perturbed metabolism in QS mutants causes physiological stress in the rice bacterial pathogen *Burkholderia glumae*. Here, we show that increased bacterial osmolality in *B. glumae* is caused by unusually high cellular concentrations of glutamate and betaine generated by QS deficiencies. QS negatively controls glutamate uptake and the expression of genes involved in the glutamine synthetase and glutamine oxoglutarate aminotransferase cycles. Thus, cellular glutamate levels were significantly higher in the QS mutants than in the wild type, and they caused hyperosmotic cellular conditions. Under the hypotonic conditions of the periplasm in the QS mutants, outer membrane bulging and vesiculation were observed, although these changes were rescued by knocking out the *gltI* gene, which encodes a glutamate transporter. Outer membrane modifications were not detected in the wild type. These results suggest that QS-dependent glutamate metabolism is critical for homeostatic osmolality. We suggest that outer membrane bulging and vesiculation might be the outcome of a physiological adaptation to relieve hypotonic osmotic stress in QS mutants. Our findings reveal how QS functions to maintain bacterial osmolality in a cooperative population.

A crowded bacterial population under limiting conditions undertakes specific sets of actions to survive in harsh environments^1^. The molecular mechanisms acting in response to diverse stresses involve specific genes or specialized systems^2^. Among the gene regulation systems that respond to physical or physiological stresses in bacteria, quorum sensing (QS), which controls sets of genes in response to population density, plays important roles^3^,^4^. In *Vibrio cholerae* and *Escherichia coli*, the QS-dependent sigma factors RpoS and σ^E^, respectively, play important roles in stress response^5^,^6^. QS controls the osmotic stress response in *Vibrio harveyi*^7^, and acyl-homoserine lactone (AHL)-mediated QS is important for the phosphate-stress response in *Pseudomonas aeruginosa* and for overcoming oxidative stress in *Burkholderia glumae* and *P. aeruginosa*^8^,^9^. Upon osmotic shifts, bacteria use osmosensing systems to regulate the expression of genes involved in the response to changes in external osmolality^10^. Previous studies have primarily focused on the mechanisms underlying the physiological and genetic responses in bacteria to external osmotic stress^11^. However, the effect of QS-mediated metabolic homeostasis on the osmotic properties of bacterial cells is poorly understood. To determine whether QS-mediated metabolic homeostasis is important for the osmotic properties of bacterial cells, we used the rice pathogen *B. glumae* strain BGR1 as a model organism because QS control of primary metabolism and metabolic fluctuations has been investigated extensively in this bacterium^12^,^13^,^14^. *B. glumae* strain BGR1 has one LuxI-R type AHL-mediated QS system, TofI-R^15^. The QS signal synthase TofI is responsible for the biosynthesis of *N*-octanoyl-homoserine lactone (C8-HSL), whose cognate receptor is TofR^15^. A complex of TofR-C8-HSL activates the expression of an IclR-type transcriptional regulator gene, *qsmR*^16^. QsmR activates the expression of genes for catalase, flagella, and oxalate biosynthesis^9^,^12^,^16^. QsmR is bifunctional because it also represses sets of genes involved in primary metabolism, including glucose uptake, substrate and oxidative phosphorylation, and *de novo* nucleotide biosynthesis^13^,^14^. Our previous metabolome analyses showed that glutamate, glutamine, and betaine were accumulated at significantly higher levels in QS mutants than in the wild type^13^ (see Supporting Information in ref. 13). Furthermore, we found outer membrane bulging and vesiculation in the QS mutants but not in the wild type of *B. glumae*. K^+^, glutamate, and betaine are major compatible solutes for osmoregulation in cellular pools^17^; therefore, we hypothesized that QS mutants might experience serious osmotic stress associated with envelope physiology.

In this study, we examined the molecular basis underlying imbalances in glutamate and glutamine levels in QS mutants and determined whether QS-mediated metabolic homeostasis is important for the maintenance of bacterial osmolality. We investigated whether such osmotic stress conditions could represent the cause of outer membrane bulging and vesiculation observed in QS mutants. We found that bacterial osmolality strongly corresponds to metabolic disorders present in *B. glumae* QS mutants and showed that outer membrane vesiculation occurs under high osmolality resulting from QS deficiency.

## Results

### QS negatively regulates glutamate uptake and nitrogen metabolism

**Figure 1:**
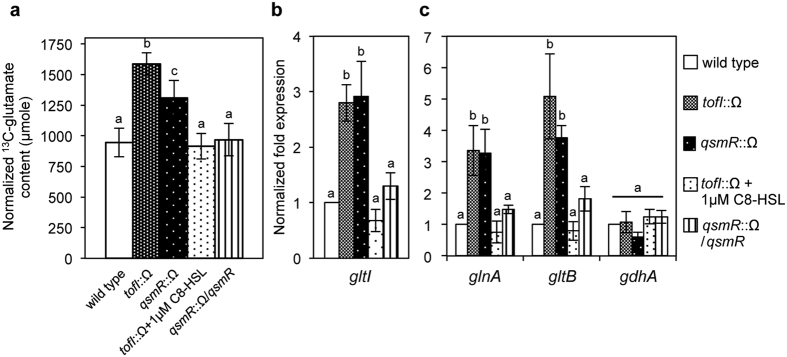
Down-regulation of glutamate uptake by QS in *B. glumae*. (**a**) Total amount of [^13^C] after the feeding of L-glutamate-3-[^13^C] in *B. glumae* wild type and QS mutants assessed by integration of the peaks generated by [^13^C]-NMR spectroscopy. (**b**,**c**) Expression levels of genes involved in glutamate transport and glutamate metabolism. The *gltI, glnA, gltB*, and *gdhA* genes encode for glutamate transporter, glutamine synthetase, glutamine oxoglutarate aminotransferase, and glutamate dehydrogenase, respectively. The gene expression levels after 10 h of incubation were quantified by qRT-PCR with three biological replicates. In (**a**,**b**) and (**c**), the given values are means ± SE. The letters (**a**,**b** and **c**) above each mean represent groupings of statistical significance based on an analysis of variance (ANOVA) and pairwise Student’s *t-*tests between strains or an ANOVA/Tukey’s correction for multiple comparisons. A value of *p* < 0.05 represented significant differences among strains.

Based on previous results showing that glutamate levels in the *tofI* mutant BGS2 and the *qsmR* mutant BGS9 were higher than that in the wild-type BGR1, we assessed the glutamate uptake ability by feeding L-glutamate-3-[^13^C] in LB broth to each of these strains. ^13^C-labeled glutamate was transported at significantly higher levels in the QS mutants than in the wild type as determined by [^13^C]-nuclear magnetic resonance (NMR) spectra (Fig. 1a; Supplementary Fig. S1). The exogenous addition of 1 μM C8-HSL to the *tofI* mutant BGS2 and genetic complementation of the *qsmR* mutant BGS9 restored the levels of glutamate uptake to that of the wild type (Fig. 1a). These results indicated that glutamate uptake is under the control of QS. Expression of *gltI* (locus ID: bglu_1g05590), which encodes a glutamate transporter, was significantly higher in the QS mutant than in the wild type (Fig. 1b), suggesting that glutamate uptake is negatively controlled by QS in *B. glumae*. In addition to differential uptake rates of glutamate between the wild type and the QS mutants, we determined whether the expression of genes involved in nitrogen metabolism is controlled by QS because glutamate levels can be influenced by the glutamine synthetase (GS) and glutamine oxoglutarate aminotransferase (GOGAT) cycles. We compared the expression levels of the *glnA* (locus ID: bglu_1g25000), *gltB* (locus ID: bglu_1g03130), and *gdhA* (locus ID: bglu_1g05580) genes, which encode GS, GOGAT, and glutamate dehydrogenase (GDH), respectively, in the wild type, the *tofI* mutant BGS2, and the *qsmR* mutant BGS9. Two genes involved in the GS/GOGAT cycle were down-regulated by QS, whereas the expression of *gdhA* was independent of QS (Fig. 1c). The expression of *gdhA* was relatively low compared with the expression levels of *glnA* and *gltB* (Fig. 1c). These results indicated that the GS/GOGAT cycle was more active in the QS mutants than in the wild type.

### QS mutants experience hyperosmotic stress

**Figure 2:**
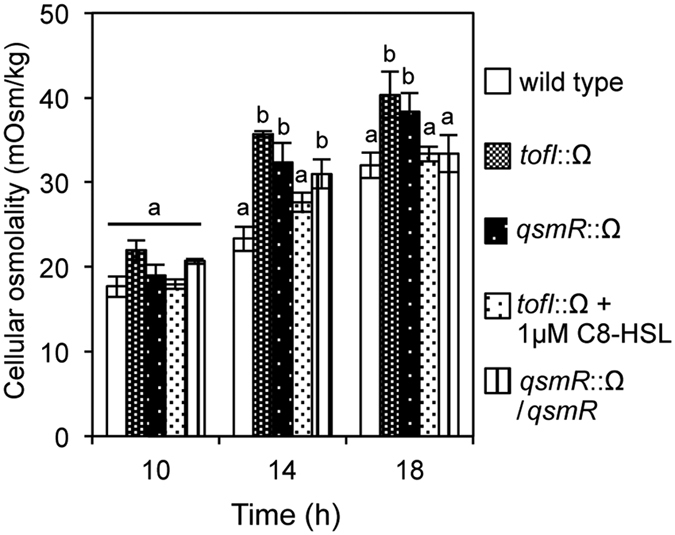
Level of cellular osmolality in QS mutants. The cellular osmolality (mOsm/kg) levels of the wild type and QS mutants were measured as described in the Methods. The error bars represent the error ranges of the experiments performed in triplicate. The letters (**a**,**b** and **c**) above each mean represent groupings of statistical significance based on an analysis of variance (ANOVA) and pairwise Student’s *t-*tests between strains or an ANOVA/Tukey’s correction for multiple comparisons. A value of *p* < 0.05 represented significant differences among strains.

To determine whether differences occur in the internal osmolality or K^+^ concentration in the *tofI* mutant BGS2 and the *qsmR* mutant BGS9 relative to that of the wild type, we measured the external and internal osmolality and K^+^ concentrations of each strain at different growth time points. No differences in internal K^+^ concentrations or external osmolality were detected among the strains (Supplementary Figs S2, S3). However, the cellular osmolality of the QS mutants was significantly higher than that of the wild type (Fig. 2). The complemented QS mutant strains exhibited a similar cellular osmolality to that of the wild type (Fig. 2). These results indicated that QS mutants experience hyperosmotic stress.

### Cell elasticity is higher in the QS mutants than the wild type

**Figure 3:**
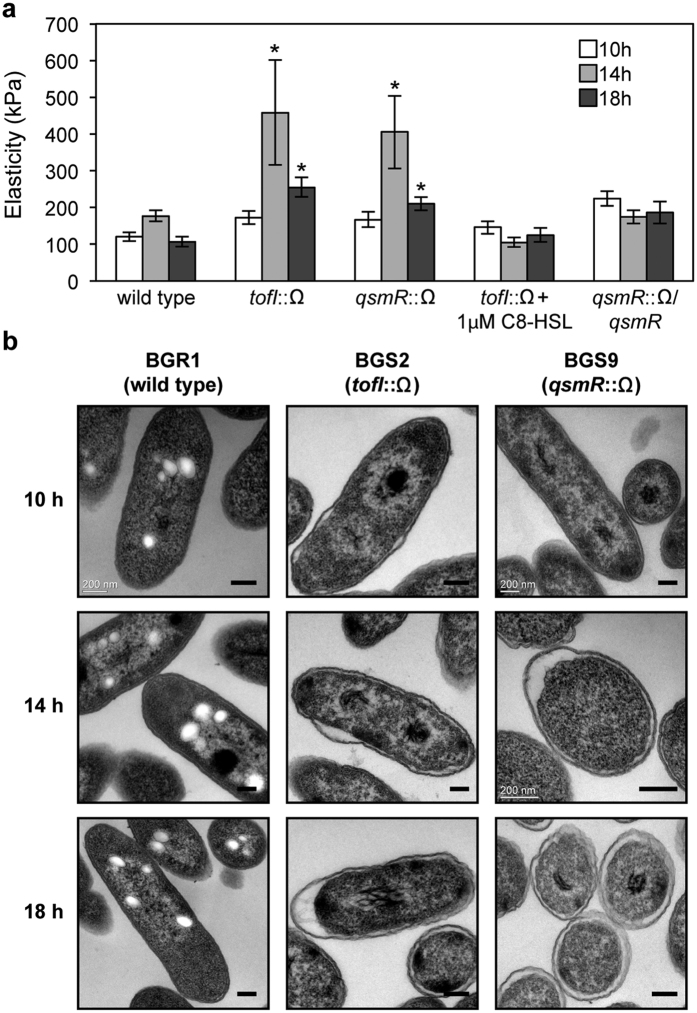
Measurement of cell elasticity and hyperhydration in the periplasm of QS mutants. (**a**) Elasticity of the wild type and QS mutant cells was measured at different time points. The elasticity (kPa) of bacterial cells was calculated via force spectra measured by AFM. The error bars represent the error ranges of the experiments obtained from at least 30 individual bacterial cells (n > 30). The asterisks represent a significant difference in elasticity (*p* < 0.05) between the wild type and QS mutants or complements as determined by ANOVA. (**b**) TEM ultrathin section microscopy images of the wild type and QS mutants at different time points are shown. All of the micrographs were selected from at least 50 pictures showing similar results. The scale bars denote 0.2 μm.

Because QS mutants accumulate more glutamate than the wild type, we hypothesized that QS mutants may experience a hypoosmotic condition resulting in water influx into the cell. To test this hypothesis, we measured the elasticity of individual cells immobilized on a surface as indicated by Young’s moduli (or elastic moduli) using atomic force microscopy (AFM) in contact mode. The cell elasticity of the QS mutants BGS2 and BGS9 was significantly higher than that of the wild type, and this result was consistent with the glutamate accumulation in the QS mutants (Fig. 3a). The QS mutants exhibited maximum cell elasticity at the late exponential stage, which was followed by a slight decrease at the stationary stage (Fig. 3a).

### Hyperhydration of the periplasm in the QS mutants

**Figure 4:**
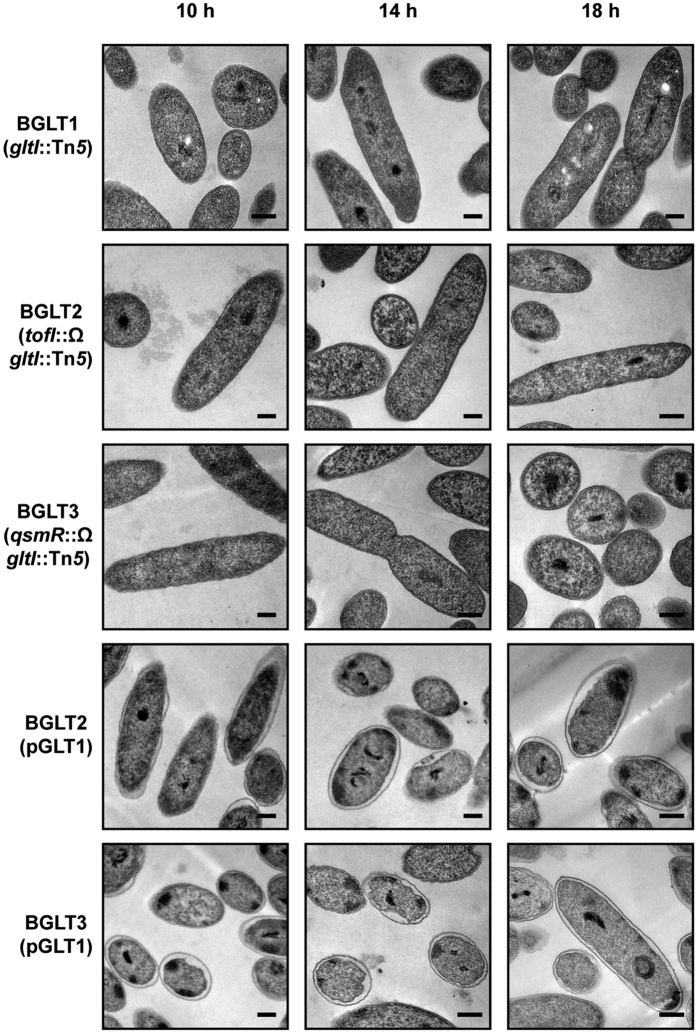
Rescue of periplasm hyperhydration by knocking out *gltI*. TEM ultrathin section micrographs of QS mutants rescued from periplasm hyperhydration by knocking out the *gltI* gene, which encodes a glutamate transporter. No hyperhydration was observed in the QS mutants carrying the *gltI* mutation. Genetic complementation using pGLT1 carrying the *gltI* gene conferred the hyperhydration phenotype to the QS mutants. The micrographs represent at least 50 images showing similar results. Scale bars indicate 0.2 μm.

**Figure 5:**
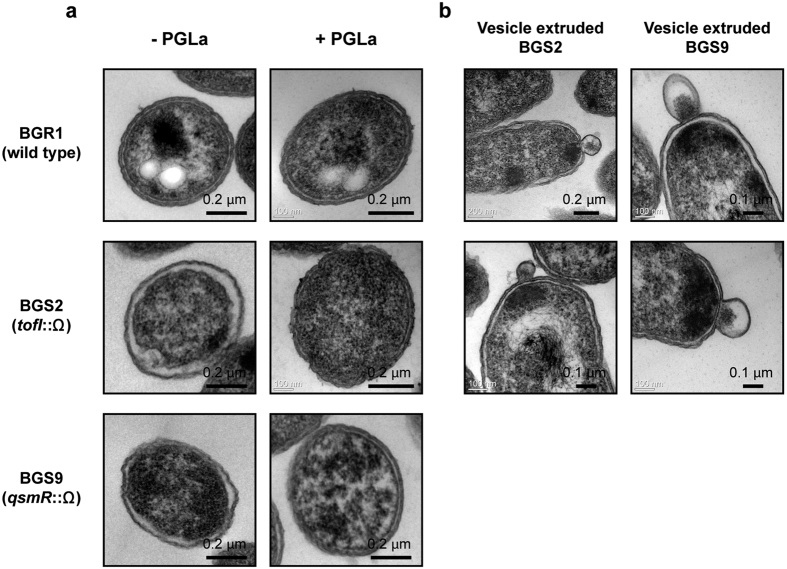
Formation of OMVs in *B. glumae* QS mutants. (**a**) TEM ultrathin section micrographs of the wild type and QS mutants grown in LB for 14 h with/without PGLa. The periplasm of the QS mutants was hyperhydrated, whereas the wild type exhibited a normal periplasm. All micrographs were selected from at least 50 pictures showing similar results. Scale bars indicate 0.2 μm. (**b**) TEM ultrathin section micrographs of the QS mutants are shown. Vesicles extruded from cell envelopes were observed after 14 h incubations in LB. The scale bars indicate 0.1 μm or 0.2 μm. OMVs were not detected in the wild type samples.

To examine the morphological changes of the QS mutants caused by hypotonic conditions, we observed ultrathin sections of QS mutant cells and wild type cells by transmission electron microscopy (TEM). The wild type cells exhibited a normal cell envelope and intact outer membrane structure during all growth stages (Fig. 3b). Conversely, the periplasmic space of 43% of the *tofI* mutant BGS2 and 36% of the *qsmR* mutant BGS9 cell populations was hyperhydrated in the late exponential phase (Fig. 3b). The exogenous addition of 1 μM C8-HSL to the *tofI* mutant BGS2 rescued the hyperhydration phenotype (Supplementary Fig. S4). In addition, hyperhydration was not observed in the *gltI* mutants of the *tofI* mutant BGS2 or the *qsmR* mutant BGS9, whereas genetic complementation with pGLT1 carrying the *gltI* gene conferred hyperhydration (Fig. 4). When the QS mutants were treated with 50 μg/ml of α-helical peptidyl-glycylleucine-carboxyamide (PGLa, an antimicrobial peptide), the number of cells possessing a hyperhydrated periplasmic space decreased to 5.1% and 4.7% in the *tofI* mutant BGS2 and *qsmR* mutant BGS9 cells, respectively, in the late exponential phase (Fig. 5a). The recovered periplasmic space and inner and outer membranes of the QS mutants appeared to be normal compared with those observed in wild type cells (Fig. 5a). However, the QS mutant cells treated with PGLa exhibited faster cell death than the untreated QS mutant cells or PGLa-treated wild type cells (Supplementary Fig. S5).

### Formation of outer membrane vesicles (OMVs) in the QS mutants

**Figure 6:**
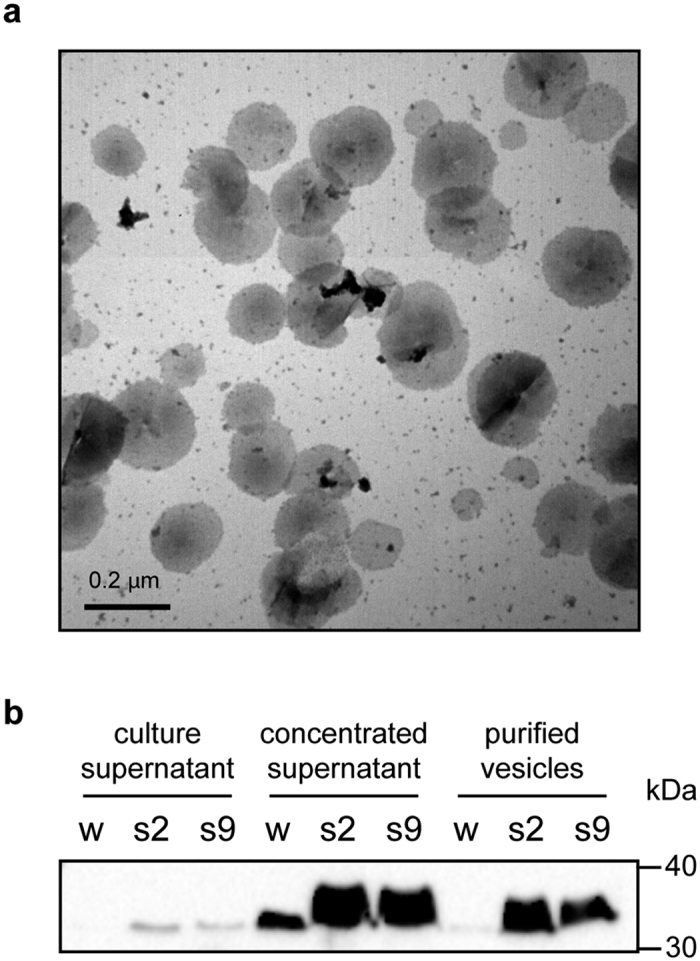
Purification of OMVs. (**a**) Uranyl acetate-stained electron micrographs of OMVs purified from the supernatant of the QS mutants by Optiprep^TM^ gradient sedimentation. The selected vesicle-containing fraction (fraction 10) was visualized by TEM. The scale bar indicates 0.2 μm. (**b**) Immunoblot detection of OmpC in OMVs extracted from the culture supernatants of the wild type (w) and the QS mutants BGS2 (s2) and BGS9 (s9) in LB after 18 h of incubation. The culture supernatant was filtered through polyvinylidene fluoride membrane filters followed by centrifugation at 110,000 × g for 2 h at 4 °C. The concentrated supernatant was resuspended with buffer. The purified OMVs were obtained by a 20–45% Optiprep^TM^density gradient. All proteins were separated by 12% SDS-PAGE.

The QS mutants exhibited a unique membranous structure that bulged from the cell envelope during the late exponential and stationary phases (Fig. 5b). We hypothesized that this structure might be isolated from the outer membrane. To determine whether the membranous bulging structure was a sign of OMV formation, the cell-free culture supernatant was fractionated by Optiprep^TM^-gradient centrifugation. Among the twelve fractions collected, the tenth fraction was chosen as a major vesicle-containing fraction as analyzed by sodium dodecyl sulfate-polyacrylamide gel electrophoresis (SDS-PAGE). An analysis of TEM micrographs of the fractions revealed numerous spherical structures ranging from 100 to 200 nm in diameter (Fig. 6a). No OMVs were detected in the cell-free supernatant or in any Optiprep^TM^ fractions prepared from the wild type. To quantify the production of OMVs in the *B. glumae* QS mutants, we assessed the amounts of OmpC, a major outer membrane protein primarily localized to the outer membrane, as a marker protein of OMVs using an anti-OmpC antibody. Immunoblot analyses revealed that OmpC was present in the cell-free culture supernatant, the culture supernatant concentrated by ultracentrifugation, and the Optiprep^TM^ fractions from the QS mutants (Fig. 6b). In the wild type samples, OmpC was not detected in the cell-free culture supernatant (Fig. 6b). Small amounts of OmpC were detected in the wild type culture supernatant concentrated by ultracentrifugation, although positive signals were not detected in the samples obtained by gradient fractionation (Fig. 6b).

### Proteomic analysis of OMVs

**Figure 7:**
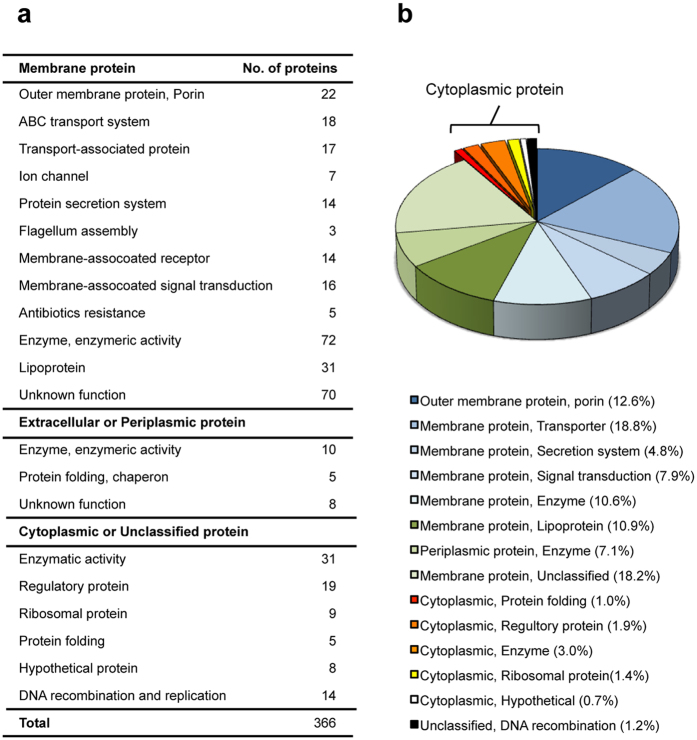
Proteomes of OMVs from the QS mutant BGS2. (**a**) Total of 366 proteins were identified in the LC-MS/MS data with high-confidence (*p* < 0.01, estimated by Proteome Discoverer^TM^, Thermo Scientific, Waltham, MA, USA) and categorized based on their subcellular localizations and functions. The subcellular localization was estimated by PSORTb version 3.02 (www.psort.org/psortb/). All of the identified and categorized proteins are listed in Supplementary Table S3. (**b**) Distribution of *B. glumae* OMV-derived proteins based on their abundance in OMVs. The abundance of each vesicular protein was estimated by peptide quantification using Proteome Discoverer^TM^.

To characterize the proteins present in the OMVs produced by the QS mutants, we analyzed the total proteomes using nano-liquid chromatography-tandem mass spectrometry (nano-LC-MS/MS). From three independent experiments, we identified a total of 366 vesicular proteins with high confidence using a highly stringent filter applied to the MS/MS spectra and categorized by subcellular localization (Supplementary Table S3). As shown in Fig. 7, 84.0%, 7.1%, and 9.2% of the proteins were classified as membrane, extracellular proteins, and cytoplasmic proteins, respectively. These results indicate that the membrane proteins identified in the *B. glumae* OMVs primarily function in the assembly of protein secretion systems, transport of specific substrates, stabilization of the outer membrane, reception of environmental signals, and degradation of specific substrates. The detected extracellular proteins were mostly associated with proteolysis, enzymatic degradation, and toluene resistance (Fig. 7a,b). The identified cytoplasmic proteins contained the major components of Gram-negative bacterial ribosomes and proteins involved in metabolic pathways, transcriptional regulation, protein folding, and DNA recombination (Fig. 7a,b).

## Discussion

Glutamate and glutamine are circulated in the central nitrogen metabolic circuit in association with three enzymes: GS, GOGAT, and GDH^18^. In enteric bacteria, internal glutamine senses external nitrogen availability, and cellular glutamate is accumulated to counteract and maintain the internal pool of K^+^ upon high osmotic shock^19^. Intensive studies on the regulation of nitrogen metabolism have identified multi-regulatory systems that respond to ammonium concentrations or other nitrogen sources^18^. However, whether QS is involved in nitrogen metabolism, especially in maintaining glutamine and glutamate pools, is not well understood. This study demonstrated that glutamate uptake and the GS/GOGAT cycles are negatively controlled by QS. Because GOGAT is the major glutamine-metabolizing enzyme, we hypothesized that glutamine levels may be high in the QS mutants. This physiological phenomenon is similar to QS-mediated metabolic slowing, as reported previously^13^. Under crowded conditions, QS functions to slow down nitrogen metabolism, as observed with carbon metabolism in *B. glumae*^13^. Therefore, metabolic slowing by QS is not limited to carbon metabolism and can be extended to nitrogen metabolism as a common phenomenon.

The uptake of K^+^ and internal glutamate accumulation are typical cellular responses upon an increase in external osmolality in bacteria^10^. Differences in internal and external K^+^ levels and the differential expression of putative genes involved in K^+^ transport were not detected between the *B. glumae* wild type and QS mutants. Changes in the osmolality of the culture medium were not observed during growth of the wild type or QS mutants. These results indicate that the observed high levels of glutamate in the QS mutants are caused by a deficiency in the slow uptake of external glutamate. Hypotonic conditions in the QS mutants induced an influx of water into the cells. Previous studies showed that mechanosensitive channels open and release water into the periplasm to avoid bursting when bacterial cells start to swell in such a hypotonic condition^20^,^21^. The impact of PGLa on the *B. glumae* QS mutants supports the finding that hyperhydration occurs in the periplasm because PGLa bound to the outer membrane surface is known to create a toroidal pore in the bacterial outer membrane^22^. As shown in Fig. 5a, the hyperhydration of the periplasm was greatly decreased in the PGLa-treated QS mutant bacterial cells. These results also indicate that hyperhydration occurred in the periplasm locally. Thus, we believe the periplasmic space in the QS mutants was hyperhydrated to supply additional osmotic pressure capacity. The periplasm of wild type *B. glumae* was not hyperhydrated. Moreover, the periplasm of the QS mutants lacking the glutamate transporter system was not hyperhydrated. These findings strongly suggest that glutamate uptake is crucial for cellular osmolality and represents a key factor affecting periplasm hyperhydration in *B. glumae*. Taken together with the finding that betaine, a major organic osmolyte, accumulates in the QS mutants^13^ (see Supporting Information in ref. 13), these results suggest that QS is involved in osmoregulation in *B. glumae*.

Another significant change observed in the QS mutants was the formation of OMVs. The observed OMV structures in the QS mutants were similar to those observed in other bacterial species^23^. The biological roles of bacterial OMVs include the modulation of the immune response in interactions with hosts^24^, trafficking of degradative enzymes^25^, trafficking of the *Pseudomonas* quinolone signal molecule^26^, and elimination of toxic materials^27^. Bacterial OMV production is a highly regulated process, and factors affecting vesiculation have been reported^24^. However, whether QS is involved in the biogenesis of OMVs is not well understood. In *P. aeruginosa*, the QS signal molecule 2-heptyl-3-hydroxy-4-quinolone (PQS) stimulates OMV formation by its direct integration into the outer membrane to induce expansion of the outer membrane leaflet^28^. However, the bilayer-couple model of OMV biogenesis in *P. aeruginosa* does not explain why *B. glumae* QS mutants produce OMVs. In the case of *Xylella fastidiosa*, the diffusible signal factor-dependent QS system strongly suppresses the release of OMVs^29^. A QS-defective mutant of *X. fastidiosa* produced more OMVs than the wild type strain and was more virulent and less adhesive to plant tissues^29^, indicating that the QS-dependent release of OMVs is related to changes of lifestyle *in planta*. However, we do not fully understand why the wild type strain of *B. glumae* does not produce OMVs under the conditions applied here. Furthermore, whether the hypotonic osmotic stress caused by QS deficiency has a direct role in OMV formation in the QS mutants is not clear. Additional direct evidence is required to demonstrate the connection between hypotonic osmotic stress and OMV biogenesis. We speculate that the secretion of OMVs in *B. glumae* QS mutants may represent a strategy to adapt to osmotic pressure resulting from internal metabolic stress.

Bacterial OMVs are composed of outer membrane and periplasmic proteins^30^. Similar to the OMVs of other Gram-negative bacteria^23^, the OMVs of *B. glumae* were enriched with membrane proteins that may have similar functions in other bacteria^24^,^25^,^26^,^27^. The cytoplasmic proteins identified in this study were primarily ribosomal or metabolic proteins. However, cytoplasmic proteins are common in other bacterial OMVs or mammalian micro-vesicles^31^,^32^. Although we cannot completely rule out the possibility of contamination with lysed cells during OMV preparation, cytoplasmic proteins can be found in the periplasmic space under osmotic stress conditions as reported in other bacteria^33^. Because the OMVs from the *B. glumae* QS mutants are produced in response to internal metabolic stresses, cytoplasmic proteins may flow into the periplasmic space in the QS mutants. Nonetheless, as with other bacterial OMVs, a clear explanation is not available for the repeated detection of these cytoplasmic proteins in the OMVs of *B. glumae*.

Notably, the OMVs from the *B. glumae* QS mutants contained chaperone and chaperonin proteins, such as DnaK, GroEL, Cpn10, Hsp90, and a putative heat shock protein. In *E. coli*, a deletion of the gene *degP*, which encodes a periplasmic protein that presents chaperone activity for misfolded proteins, resulted in the overproduction of OMVs with accumulated misfolded outer membrane proteins^34^. These results suggested that OMV production may contribute to the removal of misfolded proteins that can be toxic in the *E. coli* Δ*degP* mutant^34^. Similarly, OMVs from the *B. glumae* QS mutants may also be involved in the removal of misfolded proteins by vesiculation of the outer membrane.

Reports have proposed that OMVs from other Gram-negative bacteria contribute to lateral DNA transfer, although the mechanism of DNA packaging into OMVs is largely unknown^35^. Proteins associated with DNA recombination were identified in the OMVs from *B. glumae* QS mutants. However, DNA was not detected in these *B. glumae* OMVs.

Our results demonstrate the importance of metabolic homeostasis in maintaining bacterial osmolality in a cooperative population, and we believe that OMV formation is QS-dependent in *B. glumae*. However, whether imbalanced osmolality directly affects OMV formation at the molecular level has not been determined.

## Methods

### Bacterial strains and growth conditions

The bacterial strains and plasmids used in this study are listed in Supplementary Table S1. The *B. glumae* strains were grown in LB medium containing 0.1% (w/v) tryptone, 0.5% (w/v) yeast extract, and 0.5% (w/v) NaCl (USB, Cleveland, OH, USA) with appropriate antibiotics at 37 °C.

### Generation of S9NC5, BGLT1, BGLT2 and BGLT3

For the genetic complementation of the *qsmR* mutant BGS9, we integrated a single copy of the *qsmR* gene into the genome using mini-Tn*5* rescue mutagenesis^15^. We verified that a single copy of the *qsmR* gene was inserted into the intergenic region between bglu_1g10240 and bglu_1g10250 in chromosome 1 of BGS9, which resulted in strain S9NC5 (BGR1 *qsmR*::Ω/*qsmR*). To obtain *B. glumae gltI* mutants, we used a cosmid clone (pGLT1) containing the *gltI* (bglu_1g05590) gene and subjected it to mutagenesis using EZ-Tn*5*^TM^ <KAN-2> (Epicentre, Madison, WI, USA), as described in the manufacturer’s protocols. A single Tn*5* insertion in the *gltI* gene was marker-exchanged into the *B. glumae* strains BGR1, BGS2 (BGR1 *tofI*::Ω), and BGS9 (BGR1 *qsmR*::Ω), resulting in BGLT1, BGLT2, and BGLT3, respectively. The plasmid pGLT1 carrying the *gltI* gene was introduced into BGLT1, BGLT2, and BGLT3 for genetic complementation.

### NMR spectroscopic analysis of L-glutamate-3-[^13^C] uptake

The *B. glumae* strain BGR1 and the QS-defective mutant strains BGS2 (BGR1 *tofI*::Ω) and BGS9 (BGR1 *qsmR*::Ω) were incubated for 10 h in 10 ml of LB broth containing 0.2% (w/v) L-glutamate-3-[^13^C] (Sigma Aldrich, St. Louis, MO, USA). The colony forming units per unit volume (CFU/ml) were measured for normalization. The harvested samples were resuspended in 0.5 ml of 99% (v/v) D_2_O. As a reference, 0.5 μl of 0.5 M trimethylsilyl propanoic acid (TSP) (100 μmol) was added to each sample. Pulsed [^13^C]-NMR spectra were obtained on a Bruker Avance 600 MHz spectrometer (Bruker BioSpin Corporation, Billerica, MA, USA) with a probe temperature of 27 °C operating at 150.90 MHz with the following parameters: 30 degree pulse angle, 42.373 kHz spectral width, 65-K data points, 0.7733 s acquisition time, and 2.0 s relaxation delay. Typically, 10,240 scans were accumulated, and line broadening of 3 Hz was used during data processing. Expression levels were assigned to the integrated values of individual peaks, which were identified by reference to the chemical shift of the reference, TSP, at 0 ppm.

### Measurement of osmolality

The internal and external osmolality of the *B. glumae* strains were measured as previously described^36^. The *B. glumae* strain BGR1 and the QS-defective mutant strains BGS2 (BGR1 *tofI*::Ω) and BGS9 (BGR1 *qsmR*::Ω) were grown in LB broth for 10, 14, and 18 h at 37 °C. At each time point, the cells were harvested from the cultures, the supernatants were completely removed, and the cells were resuspended in 0.2 ml of distilled water. An equal weight of each sample was boiled for 10 min at 100 °C. The lysates were cleared by centrifugation at 10,000 × g for 20 min. To measure the external osmolality, the culture supernatants at 0, 6, 10, 14, and 18 h were collected by centrifugation (10,000 × g for 20 min). The osmolality of the culture supernatants and the cell lysates were measured with a freezing point osmometer (Osmomat 3000 Basic, Gonotec, Berlin, Germany). The instrument was calibrated using 300 and 1,000 mOsm/kg with sodium chloride solution standards.

### Analysis of internal K^+^ ions using inductively coupled plasma (ICP) emission spectrometry

The cellular K^+^ ion fluctuations in *B. glumae* during growth were measured using an ICP emission spectrometer model ICP-730ES (Varian, Mulgrave, Australia). The *B. glumae* strain BGR1 and the QS-defective mutant strains BGS2 (BGR1 *tofI*::Ω) and BGS9 (BGR1 *qsmR*::Ω) were grown in LB medium for 10 and 18 h at 37 °C. The cells were harvested and normalized by weight. The operating conditions for the instrument were set as described in the manufacturer’s manual (plasma power: 1,500 W; plasma flow: 14 l/min; auxiliary flow: 1.5 l/min; nebulizer flow: 0.7 l/min; pump rate: 15 rpm; and stabilization delay: 30 s).

### RNA isolation and quantitative reverse transcription polymerase chain reaction (qRT-PCR)

Total RNA was isolated from the *B. glumae* strains using the RNeasy mini kit (Qiagen, Venlo, Netherland) following the manufacturer’s protocol, and the isolated RNA was treated with RNase-free DNase I (Ambion, Austin, TX, USA) to remove genomic DNA. Reverse transcription was performed using 1 μg total RNA and M-MLV Reverse Transcriptase (Promega, Madison, WI, USA) for 1 h at 42 °C. The primer pairs used for qRT-PCR are listed in Table S2. Amplification of 16 S rRNA served as a positive control. The transcriptional levels were determined using SsoFast EvaGreen Supermix (Bio-Rad, Hercules, CA, USA) and the CFX96 Real-Time PCR System (Bio-Rad, Hercules, CA, USA). The thermal cycling parameters were 95 °C for 30 s, followed by 40 cycles of 95 °C for 5 s and 60 °C for 5 s. All PCR reactions were performed three times, and all data were normalized to the 16 S rRNA gene using the Bio-Rad CFX Manager software.

### Measurement of cell elasticity using AFM

The *B. glumae* strain BGR1 and the QS-defective mutant strains BGS2 (BGR1 *tofI*::Ω) and BGS9 (BGR1 *qsmR*::Ω) were grown in LB broth for 10, 14, and 18 h at 37 °C. At each time point, the cells were immobilized on poly-L-lysine-coated glass cover slips. The stiffness of the cells was measured using an AFM microscope (XE-100, Park Scientific Instrument Systems, Suwon, Korea) in contact mode. Force curves were measured on the cell surface and fit to a model of cell mechanics to extract the elasticity of bacterial cells (Young’s modulus)^37^. All force-distance curves were obtained in contact mode using v-shaped cantilevers with a pyramid-shaped Si_3_N_4_ tip (PPP-EFM-20; Park Scientific Instrument Systems, Suwon, Korea). The force spectra were recorded using the tip approach and retract speeds of 0.3 μm/s. To obtain force-distance spectra, one area of a bacterium was selected from the topographic image, and the cantilever was withdrawn by 3–10 μm. The AFM tip was then lowered manually by a piezoelectric actuator to the surface in 1 μm increments until deflection of the cantilever was observed. The force spectra were recorded for at least 30 bacterial cells from each sample. The recorded force spectra were analyzed to determine Young’s moduli (the elasticity moduli) of cells using XEI software (version 1.8.0, Park Scientific Instrument Systems, Suwon, Korea).

### Transmission electron microscopy (TEM)

The *B. glumae* strain BGR1, the QS-defective mutant strains BGS2 (BGR1 *tofI*::Ω) and BGS9 (BGR1 *qsmR*::Ω), and the *gltI* mutants of the wild type (BGLT1) and QS mutants (BGLT2 and BGLT3) were grown for 10, 14, and 18 h at 37 °C, harvested by centrifugation at 10,000 × g for 10 min, fixed with 2.5% glutaraldehyde for 2 h, post-fixed in 1% osmium tetroxide for 2 h, dehydrated in a graded ethanol series (30–100%), and embedded in Spurr’s resin. Ultrathin sections were prepared using an ultramicrotome (EM UC7, Leica, Wetzlar, Germany) and placed on 150-mesh coated copper grids followed by staining with 2% uranyl acetate and lead citrate. For the TEM analysis of the OMVs, purified vesicles were applied to 150-mesh copper grids and negatively stained with 2% uranyl acetate. The electron micrographs were acquired and recorded using the JEM 1010 microscope (JEOL, Tokyo, Japan) at an acceleration voltage of 80 kV and the LIBRA 120 energy-filtrating microscope (Carl Zeiss, Oberkochen, Germany) at 100 kV.

### Immunoblotting using an anti-OmpC antibody

Supernatants, resuspended OMV-containing pellets, and OMVs purified by density-gradient centrifugation from the *B. glumae* strain BGR1 and the QS-defective mutant strains BGS2 (BGR1 *tofI*::Ω) and BGS9 (BGR1 *qsmR*::Ω) were separated by 12% SDS-PAGE and transferred to a polyvinylidene fluoride (PVDF) membrane. The blocked membrane was incubated with a rabbit polyclonal antibody against *B. glumae* outer membrane protein C (OmpC). The membrane was then probed with a rabbit IgG-HRP antibody (Life Technologies, Carlsbad, CA, USA), which was followed by visualization with a chemiluminescent substrate (Bio-Rad, Hercules, CA, USA).

### Proteomic analysis

To assess the proteomic profiles of the OMVs from *B. glumae* QS-deficient mutants, we performed in-solution and in-gel trypsin digestion and then LC-MS/MS analyses using a high-resolution LC tandem mass spectrometer (Q-TOF 5600, AB SCIEX, Framingham, MA, USA) at least three times. All MS/MS spectra were processed using Proteome Discoverer^TM^ (Thermo Scientific, Waltham, MA, USA). The peptides possessing at least six amino acids were considered for identification. The false discovery rates for both peptides and proteins were set at 0.01. The proteins were identified using the *B. glumae* BGR1 genome database (GenBank accession numbers: CP001503–CP001508), and their subcellular localizations were predicted based on PSORTb version 3.02 (www.psort.org/psortb/).

### Isolation and purification of OMVs

OMVs from *B. glumae* were isolated and prepared according to previously described protocols^38^. The culture supernatant was filtered through a 0.45-μm PVDF membrane filter (Millipore, Billerica, MA, USA) followed by centrifugation at 110,000 × g for 2 h at 4 °C. The resulting OMV-containing pellet was resuspended with Dulbecco’s phosphate buffered saline (DPBS) buffer. The resuspended pellets were loaded on a 20–45% Optiprep^TM^ (Sigma Aldrich, St. Louis, MO, USA) gradient and centrifuged at 110,000 × g for 20 h at 4 °C. Twelve fractions were collected and analyzed by 12% SDS-PAGE to determine the OMV-containing fraction. The purified OMVs were stored at 4 °C or −80 °C until use in immunoblot assays and proteomic analyses.

### Statistical analysis

All statistical analyses and ANOVA testing followed by Tukey’s HSD post hoc analysis were performed using IBM SPSS Statistics software (version 20 x86-x64; IBM corp., Armonk, NY, USA).

## Additional Information

**How to cite this article:** Kang, Y. *et al*. Critical role of quorum sensing-dependent glutamate metabolism in homeostatic osmolality and outer membrane vesiculation in *Burkholderia glumae. Sci. Rep.* **7**, 44195; doi: 10.1038/srep44195 (2017).

**Publisher's note:** Springer Nature remains neutral with regard to jurisdictional claims in published maps and institutional affiliations.

## Supplementary Material

Supplementary Information
