# Supplementary material for: Critical role of quorum sensing-dependent glutamate metabolism in homeostatic osmolality and outer membrane vesiculation in Burkholderia glumae
Source: Sci Rep. 2017 Mar 8;7:44195. doi: 10.1038/srep44195 (PMC5341150; doi:10.1038/srep44195)
Supplement: Supplementary Information [file srep44195-s1.pdf]

Supplementary Information

Critical role of quorum sensing-dependent glutamate metabolism in homeostatic osmolality and outer membrane vesiculation in *Burkholderia glumae*

Yongsung Kang,[1] Eunhye Goo,[1] Jinwoo Kim,[2] and Ingyu Hwang[1]*

[1]Department of Agricultural Biotechnology, Seoul National University, Seoul 08826, Republic of Korea

[2]Division of Applied Life Science and Institute of Agriculture and Life Sciences, Gyeongsang National University, Jinju 52828, Republic of Korea

*Corresponding author
E-mail: ingyu@snu.ac.kr
Tel: +82-2-880-4676
Fax: +82-2-873-2317

Figure S1.

The uptake of L-glutamate-3-[^{13}C] in *B. glumae* wild type (BGR1), the *tofI* mutant (BGS2), the *qsmR* mutant (BGS9), BGS2 supplemented with C8-HSL (BGS2+C8-HSL), and the genetically complemented BGS9 (S9NC5). The level of L-glutamate-3-[^{13}C] was measured using [^{13}C]-nuclear magnetic resonance spectroscopy.

1

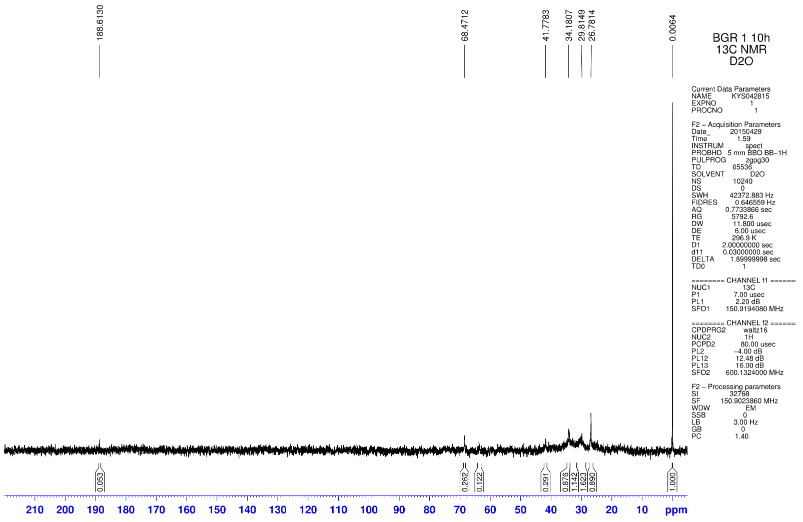
188.6130
68.4712
41.7783
34.1807
29.8149
26.7814
0.0064
BGR 1 10h
13C NMR
D2O
Current Data Parameters
NAME KYS042815
EXPNO 1
PROCNO 1
F2 – Acquisition Parameters
Date_ 20150429
Time 1.59
INSTRUM spect
PROBHD 5 mm BBO BB–1H
PULPROG zgpg30
TD 65536
SOLVENT D2O
NS 10240
DS 0
SWH 42372.883 Hz
FIDRES 0.646559 Hz
AQ 0.7733866 sec
RG 5792.6
DW 11.800 usec
DE 6.00 usec
TE 296.9 K
D1 2.00000000 sec
d11 0.03000000 sec
DELTA 1.89999998 sec
TD0 1
======== CHANNEL f1 ========
NUC1 13C
P1 7.00 usec
PL1 2.20 dB
SFO1 150.9194080 MHz
======== CHANNEL f2 ========
CPDPRG2 waltz16
NUC2 1H
PCPD2 80.00 usec
PL2 –4.00 dB
PL12 12.48 dB
PL13 16.00 dB
SFO2 600.1324000 MHz
F2 – Processing parameters
SI 32768
SF 150.9023860 MHz
WDW EM
SSB 0
LB 3.00 Hz
GB 0
PC 1.40
ppm

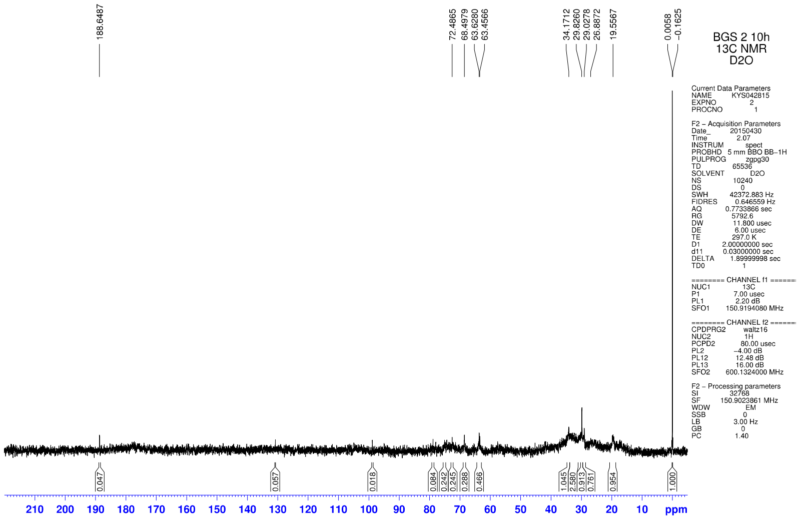
188.6487
72.4865
68.4979
63.6280
63.4566
34.1712
29.8260
29.0278
26.8872
19.5567
0.0058
–0.1625
BGS 2 10h
13C NMR
D2O
Current Data Parameters
NAME KYS042815
EXPNO 2
PROCNO 1
F2 – Acquisition Parameters
Date_ 20150430
Time 2.07
INSTRUM spect
PROBHD 5 mm BBO BB–1H
PULPROG zgpg30
TD 65536
SOLVENT D2O
NS 10240
DS 0
SWH 42372.883 Hz
FIDRES 0.646559 Hz
AQ 0.7733866 sec
RG 5792.6
DW 11.800 usec
DE 6.00 usec
TE 297.0 K
D1 2.00000000 sec
d11 0.03000000 sec
DELTA 1.89999998 sec
TD0 1
======== CHANNEL f1 ========
NUC1 13C
P1 7.00 usec
PL1 2.20 dB
SFO1 150.9194080 MHz
======== CHANNEL f2 ========
CPDPRG2 waltz16
NUC2 1H
PCPD2 80.00 usec
PL2 –4.00 dB
PL12 12.48 dB
PL13 16.00 dB
SFO2 600.1324000 MHz
F2 – Processing parameters
SI 32768
SF 150.9023861 MHz
WDW EM
SSB 0
LB 3.00 Hz
GB 0
PC 1.40
ppm

2

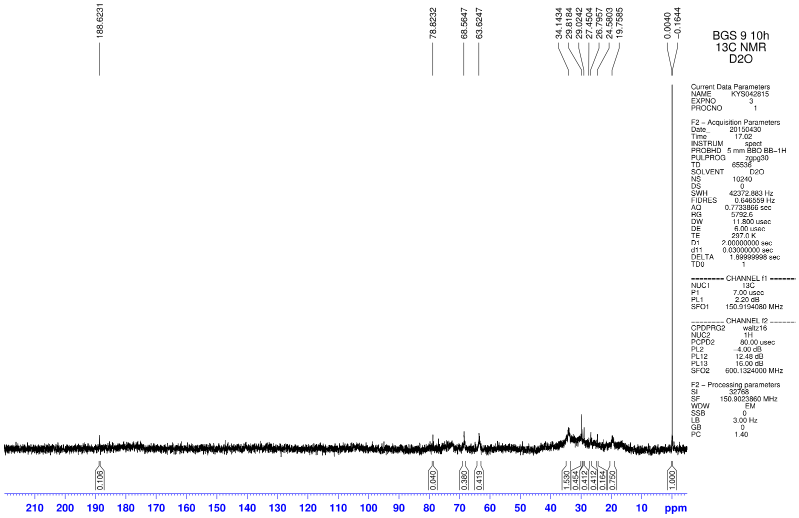

188.6231
78.8232
68.5647
63.6247
34.1434
29.8184
29.0242
27.4504
26.7957
24.5803
19.7585
0.0040
-0.1644
BGS 9 10h
13C NMR
D2O
Current Data Parameters
NAME KYS042815
EXPNO 3
PROCNO 1
F2 - Acquisition Parameters
Date_ 20150430
Time 17.02
INSTRUM spect
PROBHD 5 mm BBO BB-1H
PULPROG zgpg30
TD 65536
SOLVENT D2O
NS 10240
DS 0
SWH 42372.883 Hz
FIDRES 0.646559 Hz
AQ 0.7733866 sec
RG 5792.6
DW 11.800 usec
DE 6.00 usec
TE 297.0 K
D1 2.00000000 sec
d11 0.03000000 sec
DELTA 1.89999998 sec
TD0 1
======== CHANNEL f1 ========
NUC1 13C
P1 7.00 usec
PL1 2.20 dB
SFO1 150.9194080 MHz
======== CHANNEL f2 ========
CPDPRG2 waltz16
NUC2 1H
PCPD2 80.00 usec
PL2 -4.00 dB
PL12 12.48 dB
PL13 16.00 dB
SFO2 600.1324000 MHz
F2 - Processing parameters
SI 32768
SF 150.9023860 MHz
WDW EM
SSB 0
LB 3.00 Hz
GB 0
PC 1.40
0.106
0.040
0.380
0.419
1.530
0.454
0.412
0.412
0.184
0.750
1.000
210 200 190 180 170 160 150 140 130 120 110 100 90 80 70 60 50 40 30 20 10 ppm

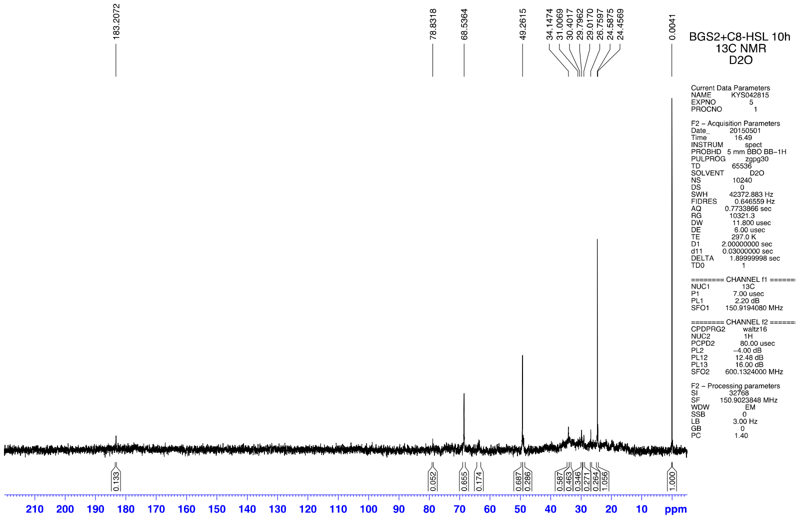

183.2072
78.8318
68.5364
49.2615
34.1474
31.0069
30.4017
29.7962
29.0170
26.7597
24.5875
24.4569
0.0041
BGS2+C8-HSL 10h
13C NMR
D2O
Current Data Parameters
NAME KYS042815
EXPNO 5
PROCNO 1
F2 - Acquisition Parameters
Date_ 20150501
Time 16.49
INSTRUM spect
PROBHD 5 mm BBO BB-1H
PULPROG zgpg30
TD 65536
SOLVENT D2O
NS 10240
DS 0
SWH 42372.883 Hz
FIDRES 0.646559 Hz
AQ 0.7733866 sec
RG 10321.3
DW 11.800 usec
DE 6.00 usec
TE 297.0 K
D1 2.00000000 sec
d11 0.03000000 sec
DELTA 1.89999998 sec
TD0 1
======== CHANNEL f1 ========
NUC1 13C
P1 7.00 usec
PL1 2.20 dB
SFO1 150.9194080 MHz
======== CHANNEL f2 ========
CPDPRG2 waltz16
NUC2 1H
PCPD2 80.00 usec
PL2 -4.00 dB
PL12 12.48 dB
PL13 16.00 dB
SFO2 600.1324000 MHz
F2 - Processing parameters
SI 32768
SF 150.9023848 MHz
WDW EM
SSB 0
LB 3.00 Hz
GB 0
PC 1.40
0.133
0.052
0.655
0.174
0.687
0.286
0.587
0.463
0.346
0.271
0.264
1.056
1.000
210 200 190 180 170 160 150 140 130 120 110 100 90 80 70 60 50 40 30 20 10 ppm

3

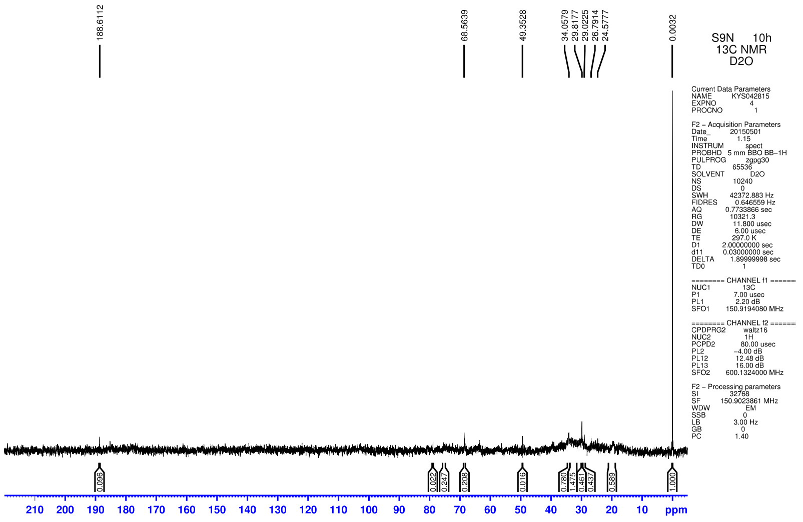
188.6112
68.5639
49.3528
34.0579
29.8177
29.0225
26.7914
24.5777
0.0032
S9N 10h
13C NMR
D2O
Current Data Parameters
NAME KYS042815
EXPNO 4
PROCNO 1
F2 – Acquisition Parameters
Date_ 20150501
Time 1.15
INSTRUM spect
PROBHD 5 mm BBO BB−1H
PULPROG zgpg30
TD 65536
SOLVENT D2O
NS 10240
DS 0
SWH 42372.883 Hz
FIDRES 0.646559 Hz
AQ 0.7733866 sec
RG 10321.3
DW 11.800 usec
DE 6.00 usec
TE 297.0 K
D1 2.00000000 sec
d11 0.03000000 sec
DELTA 1.89999998 sec
TD0 1
======== CHANNEL f1 ========
NUC1 13C
P1 7.00 usec
PL1 2.20 dB
SFO1 150.9194080 MHz
======== CHANNEL f2 ========
CPDPRG2 waltz16
NUC2 1H
PCPD2 80.00 usec
PL2 −4.00 dB
PL12 12.48 dB
PL13 16.00 dB
SFO2 600.1324000 MHz
F2 – Processing parameters
SI 32768
SF 150.9023861 MHz
WDW EM
SSB 0
LB 3.00 Hz
GB 0
PC 1.40
210 200 190 180 170 160 150 140 130 120 110 100 90 80 70 60 50 40 30 20 10 ppm

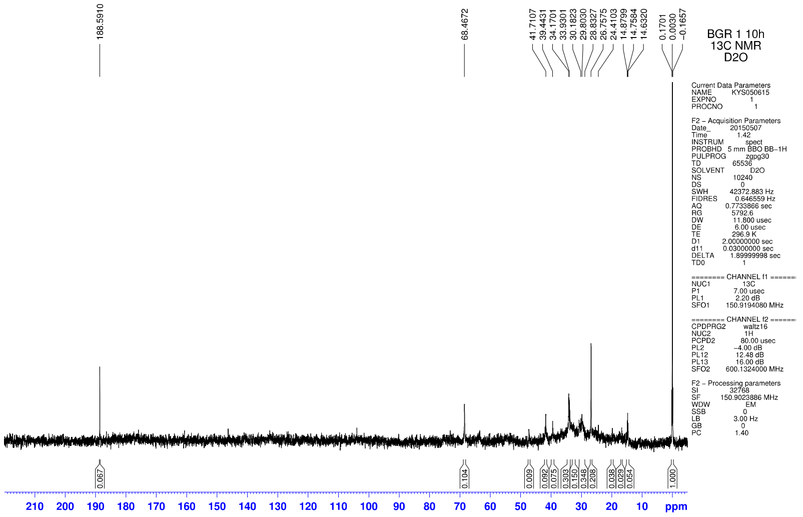
188.5910
68.4672
41.7107
39.4431
34.1701
33.9301
30.1823
29.8030
28.8327
26.7575
24.4103
14.8799
14.7584
14.6320
0.1701
0.0030
−0.1657
BGR 1 10h
13C NMR
D2O
Current Data Parameters
NAME KYS050615
EXPNO 1
PROCNO 1
F2 – Acquisition Parameters
Date_ 20150507
Time 1.42
INSTRUM spect
PROBHD 5 mm BBO BB−1H
PULPROG zgpg30
TD 65536
SOLVENT D2O
NS 10240
DS 0
SWH 42372.883 Hz
FIDRES 0.646559 Hz
AQ 0.7733866 sec
RG 5792.6
DW 11.800 usec
DE 6.00 usec
TE 296.9 K
D1 2.00000000 sec
d11 0.03000000 sec
DELTA 1.89999998 sec
TD0 1
======== CHANNEL f1 ========
NUC1 13C
P1 7.00 usec
PL1 2.20 dB
SFO1 150.9194080 MHz
======== CHANNEL f2 ========
CPDPRG2 waltz16
NUC2 1H
PCPD2 80.00 usec
PL2 −4.00 dB
PL12 12.48 dB
PL13 16.00 dB
SFO2 600.1324000 MHz
F2 – Processing parameters
SI 32768
SF 150.9023886 MHz
WDW EM
SSB 0
LB 3.00 Hz
GB 0
PC 1.40
210 200 190 180 170 160 150 140 130 120 110 100 90 80 70 60 50 40 30 20 10 ppm

4

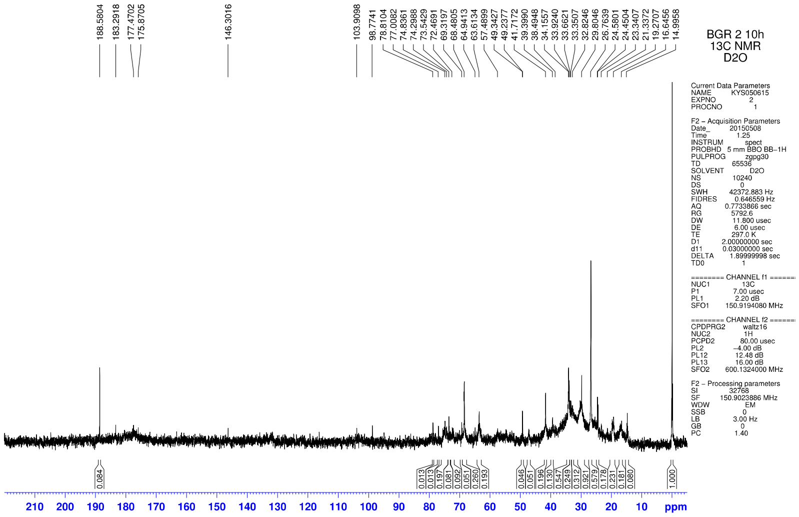

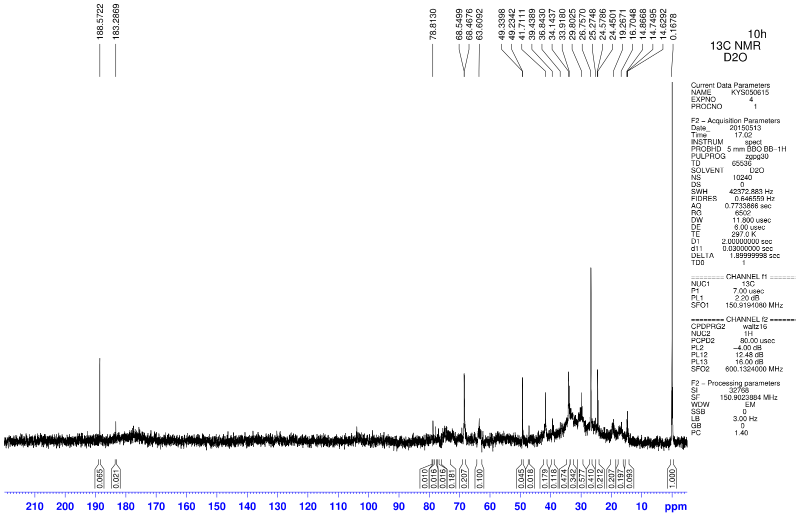

5

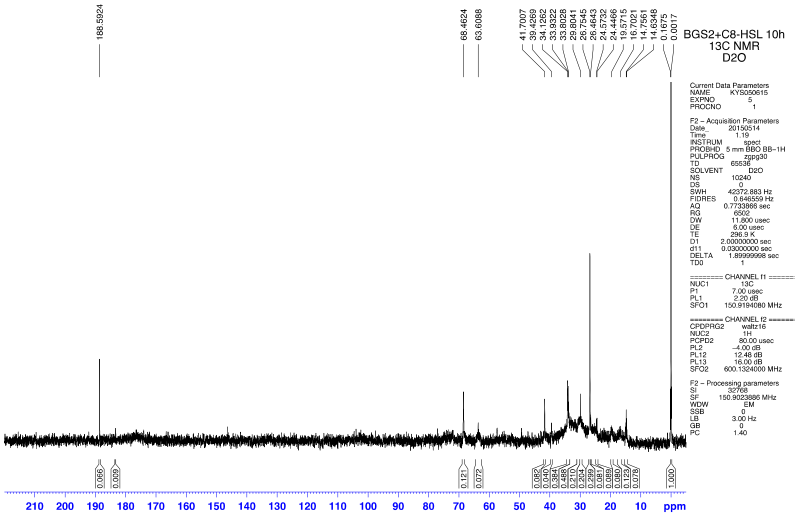

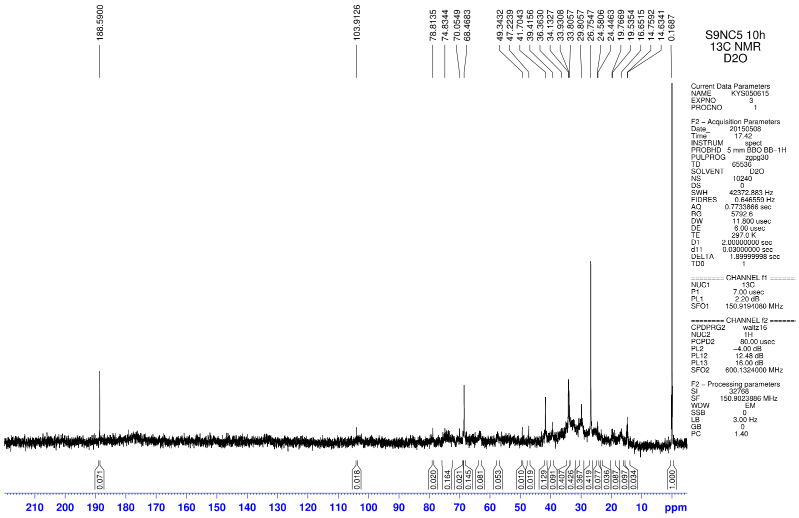

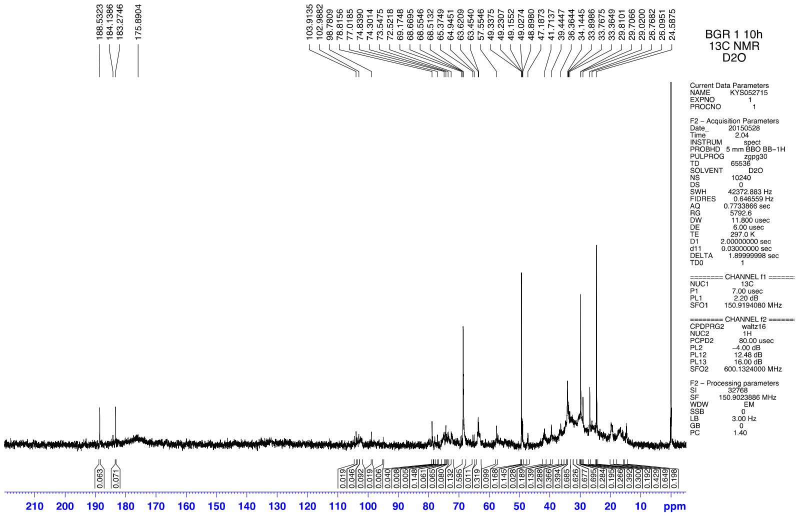
BGR 1 10h
13C NMR
D2O

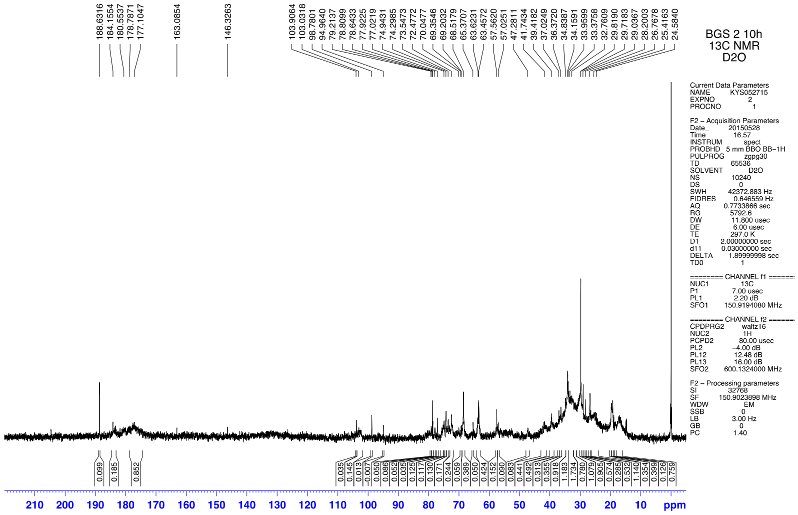
BGS 2 10h
13C NMR
D2O

7

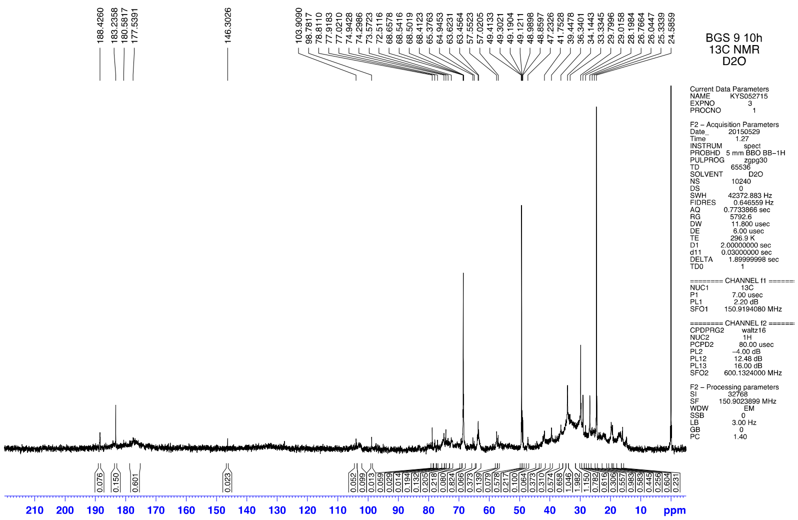

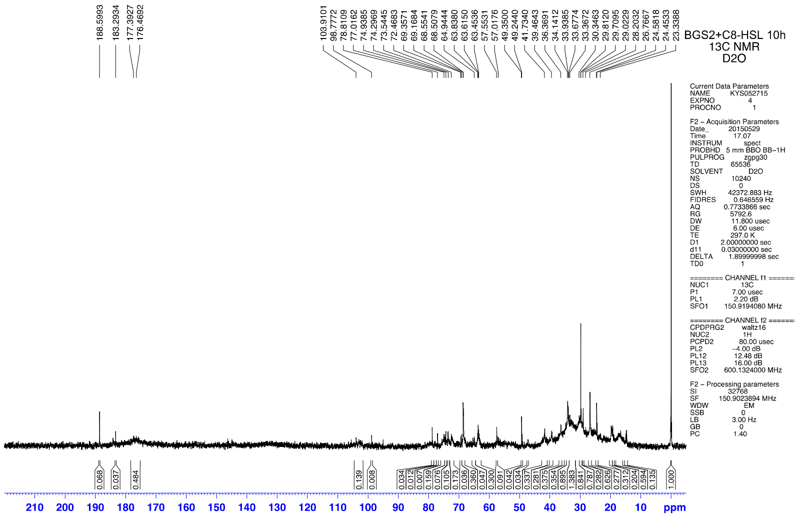

8

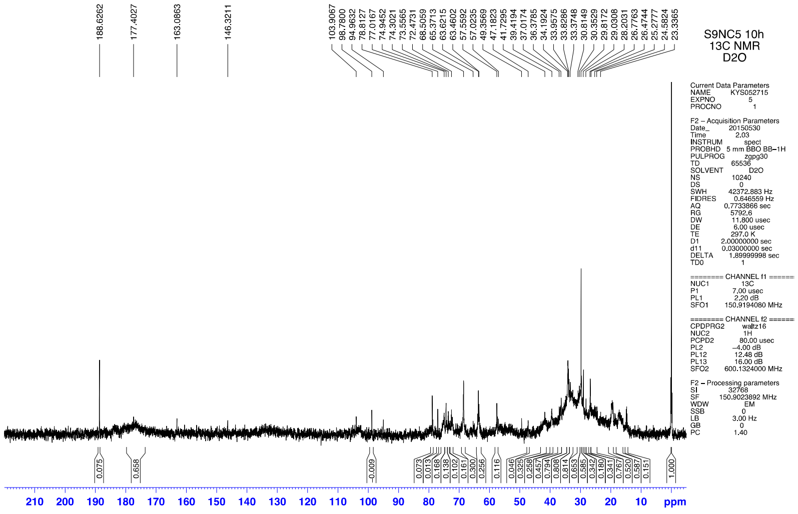
188.6262
177.4027
163.0863
146.3211
103.9067
98.7800
94.9632
78.8127
77.0167
74.9452
74.3021
73.5565
72.4731
68.5059
65.3713
63.6215
63.4602
57.5592
57.0235
49.3569
47.1823
41.7295
39.4194
37.0174
36.3785
34.1924
33.9575
33.8286
33.3748
30.8149
30.3529
29.8172
29.0308
28.2031
26.7763
26.4744
25.2777
24.5824
23.3365
S9NC5 10h
13C NMR
D2O
Current Data Parameters
NAME KYS052715
EXPNO 5
PROCNO 1
F2 – Acquisition Parameters
Date_ 20150530
Time 2.03
INSTRUM spect
PROBHD 5 mm BBO BB–1H
PULPROG zgpg30
TD 65536
SOLVENT D2O
NS 10240
DS 0
SWH 42372.883 Hz
FIDRES 0.646559 Hz
AQ 0.7733866 sec
RG 5792.6
DW 11.800 usec
DE 6.00 usec
TE 297.0 K
D1 2.00000000 sec
d11 0.03000000 sec
DELTA 1.89999998 sec
TD0 1
======== CHANNEL f1 ======
NUC1 13C
P1 7.00 usec
PL1 2.20 dB
SFO1 150.9194080 MHz
======== CHANNEL f2 ======
CPDPRG2 waltz16
NUC2 1H
PCPD2 80.00 usec
PL2 –4.00 dB
PL12 12.48 dB
PL13 16.00 dB
SFO2 600.1324000 MHz
F2 – Processing parameters
SI 32768
SF 150.9023892 MHz
WDW EM
SSB 0
LB 3.00 Hz
GB 0
PC 1.40
210 200 190 180 170 160 150 140 130 120 110 100 90 80 70 60 50 40 30 20 10 ppm

Figure S2.

The internal potassium ion (K^+) levels in wild type (BGR1), the *tofI* mutant (BGS2), the *qsmR* mutant (BGS9), and BGS2 strains complemented with C8-HSL. The levels of internal K^+ (mg/kg) were measured from *B. glumae* strains cultured in LB medium for 10 h and 18 h. All samples were normalized by weight of cells. The error bars represent the standard error ranges of the triplicate experiments.

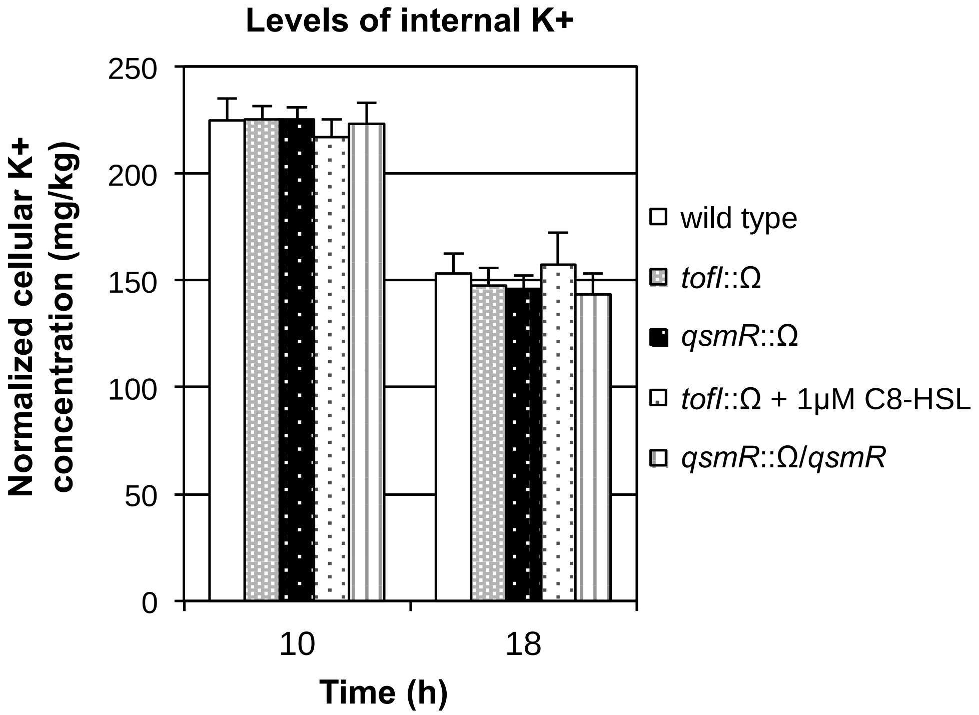

Figure S3.

The osmolality (mOsm/kg) levels in bacterial culture media during growth. The strains of *B. glumae* wild type, the *tofI* mutant (BGS2), the *qsmR* mutant (BGS9), BGS2 complemented with C8-HSL, and genetically complemented BGS9 (S9NC5) were grown in LB medium for 0 h, 6 h, 10 h, 14 h, and 18 h. The external osmolality was measured from the culture fluid of each strain. The error bars represent the standard error ranges of the triplicate experiments.

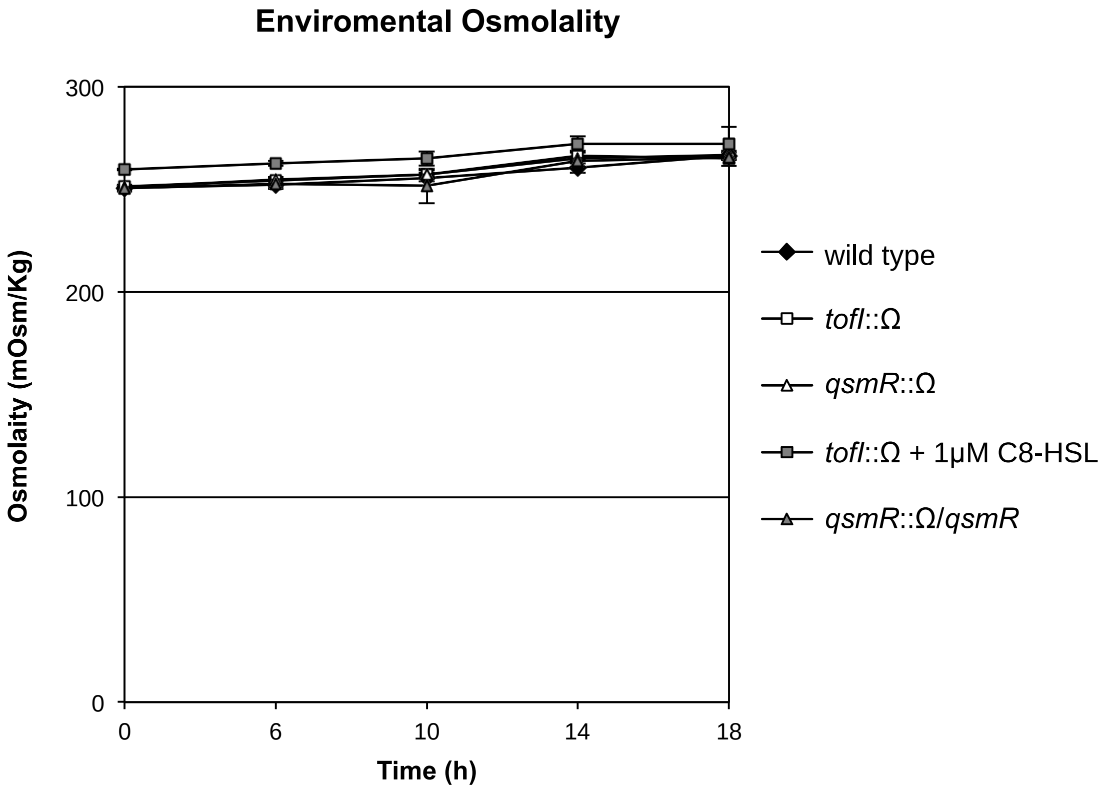

Figure S4.

TEM ultrathin section micrographs of rescued normal periplasm from the *tofI* mutant BGS2 supplemented with 1 μM C8-HSL. The micrographs represent at least 50 images showing similar results. The scale bars indicate 0.5 μm.

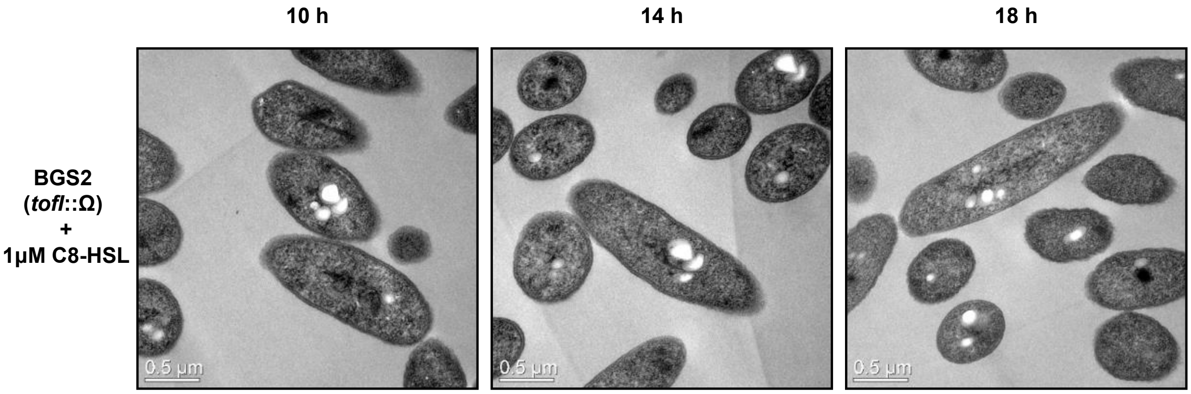

Figure S5.

Growth of the *B. glumae* wild type (BGR1), the *tofI* mutant (BGS2), and the *qsmR* mutant (BGS9) in the (a) presence or (b) absence of PGLa (50 μg/ml). The error bars indicate the standard error ranges of the triplicate experiments.

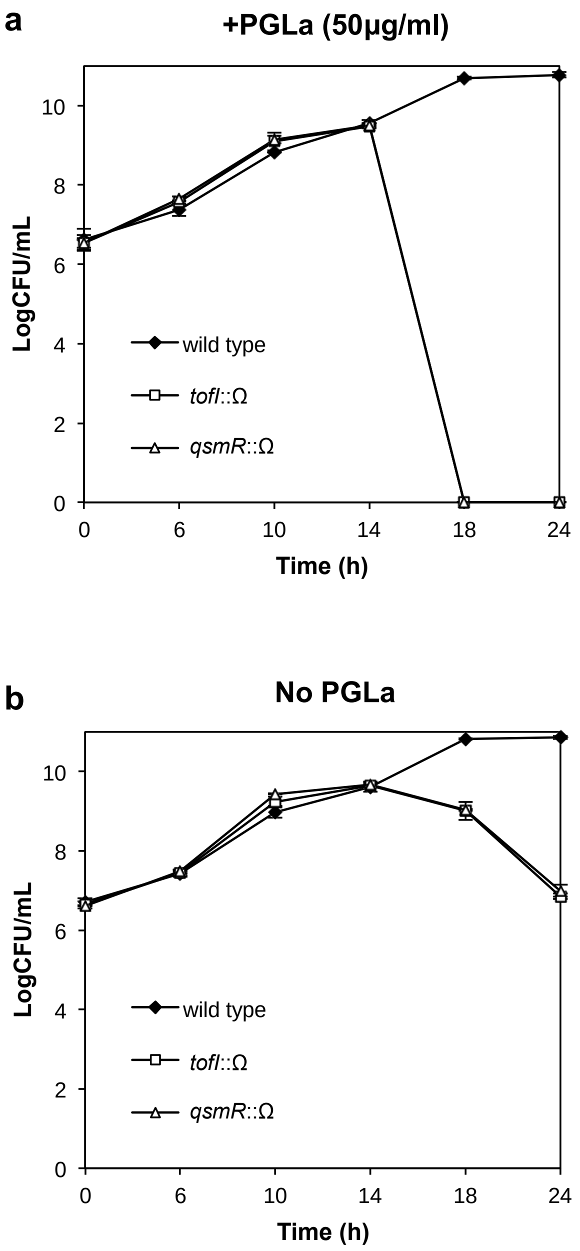

Table S1.

The bacterial strains used in this study.

Strain or plasmid	Characteristics	Source or reference
Escherichia coli		
DH5α	F^- Φ80d*lacZ* ΔM15 (*lacZYA-argF*) *U169 recA1 endA1 hsdR17*($r_K^+m_K^+$) *supE44 thi-1 gyrA relA1*	Gibco BRL
S17-1	Tra^+ *recA* Sp^R	Simon *et al*., 1983
Burkholderia glumae		
BGR1	Wild type, Rif^R	Kim *et al*., 2004
BGS2	BGR1 *tofI*::Ω	Kim *et al*., 2004
BGS9	BGR1 *qsmR*::Ω	Kim *et al*., 2007
S9NC5	BGR1 *qsmR*::Ω/*qsmR*	This study
BGLT1	BGR1 *gltI*::Tn*5*	This study
BGLT2	BGS2 *gltI*::Tn*5*	This study
BGLT3	BGS9 *gltI*::Tn*5*	This study
Plasmid		
pLAFR3	Tra^-, Mob^+, RK2 replicon, Tet^R	Staskawicz *et al*., 1987
pGLT1	23.5 kb DNA fragment including the *gltI* gene from strain BGR1 cloned into pLAFR3	This study

Table S2.

List of the oligonucleotide primers used in this study.

Primer name[a]	Sequence (5′ to 3′)
GOGAT-L	CAAGGAGCTGAAGGACAACC
GOGAT-R	GAGCGTCTTGTTCTTGTTCG
GDH-L	GGATGTCGGTCAAGAATGC
GDH-R	TGGTTCATCGAGTAGGTGTCC
GS-L	GACACGTTCCAGGACATGC
GS-R	CCAGATCGACTGGTGAACG
GTR-L	CAGAACCAGCAGGTGATCG
GTR-R	AATCCTTCGAGGTCATCAGG

a: L, forward primer; R, reverse primer

Table S3.

List of the proteins identified in *B. glumae* OMVs.

Accession[a]	Description	Score[b]	Coverage[b]	Subcellular localization[c]
**Outer membrane protein, porin**				
bglu_1g02240	Putative outer membrane protein	7.28	17.99	outer
bglu_1g03110	Outer membrane protein, OmpW family	62.67	37.86	outer
bglu_1g06700	OmpA domain-containing protein	363.47	73.53	outer
bglu_1g08340	Porin	560.34	56.66	outer
bglu_1g08710	OmpA/MotB domain-containing protein	625.61	29.36	outer
bglu_1g11700	Outer membrane protein V	2.07	5.02	inner
bglu_1g12810	Outer membrane protein, OMP85 family	404.99	47.79	outer
bglu_1g16050	OmpW family protein	1881.63	79.34	outer
bglu_1g21740	Porin	10.15	7.10	inner
bglu_1g27820	Outer membrane protein, porin	82.48	29.09	outer
bglu_1g28020	OmpA family protein	250.82	38.36	outer
bglu_1g33010	Outer membrane porin	44.03	24.47	outer
bglu_2g02010	Outer membrane porin, OmpC	79.18	29.43	outer

	family			
bglu_2g05080	Outer membrane protein (porin)	3.73	6.05	outer
bglu_2g06350	Putative outer membrane protein OprM precursor	439.41	62.25	periplasm
bglu_2g08600	Outer membrane porin	465.08	41.71	outer
bglu_2g12390	Porin, Gram-negative type	10.92	5.69	outer
bglu_2g14610	Porin	3083.59	70.64	outer
bglu_2g15000	Outer membrane porin OpcP	2.15	2.12	outer
bglu_2g21350	Carbohydrate porin, OprB family	9.48	12.07	inner
bglu_2g22070	Outer membrane porin, OprD family	9.63	7.02	inner
bglu_2g22280	Porin, Gram-negative type	46.31	23.14	outer
**Membrane protein, Transporter**				
bglu_1g00600	Branched-chain amino acid ABC transporter, periplasmic branched-chain amino acid-binding protein	5.81	9.45	inner
bglu_1g00730	ATP synthase F0, beta subunit	6.92	21.15	inner
bglu_1g00740	ATP synthase subunit D	10.23	15.64	cytoplasm
bglu_1g00750	ATP synthase subunit A	67.91	22.61	cytoplasm
bglu_1g00760	ATP Synthase gamma chain	2.06	4.11	cytoplasm
bglu_1g00770	ATP synthase F1, beta subunit	45.55	22.84	cytoplasm

bglu_1g02040	amino acid ABC transporter periplasmic amino acid-binding protein	1162.15	55.53	periplasm
bglu_1g02160	Transporter	59.24	16.67	periplasm
bglu_1g03060	Glycerol-3-phosphate ABC transporter, periplasmic glycerol-3-phosphate-binding protein	46.04	24.04	inner
bglu_1g05590	glutamate/aspartate ABC transporter periplasmic glutamate/aspartate-binding protein	141.57	36.36	periplasm
bglu_1g06690	tol-pal system protein YbgF	286.66	56.63	periplasm
bglu_1g06710	translocation protein TolB	871.86	69.77	outer
bglu_1g06740	MotA/TolQ/ExbB proton channel	4.85	6.22	inner
bglu_1g07020	Bacterial extracellular solute-binding protein	5.28	12.03	inner
bglu_1g07070	Transport-associated protein	106.96	45.08	periplasm
bglu_1g08070	ABC-type sugar transport system periplasmic component	164.29	46.99	periplasm
bglu_1g08080	Binding-protein-dependent transport systems inner membrane component	2.08	3.83	cytoplasm
bglu_1g08100	ABC transporter, carbohydrate uptake transporter-1 (CUT1)	2.52	4.57	inner

	family, ATP-binding protein			
bglu_1g10160	amino acid ABC transporter periplasmic amino acid-binding protein	33.06	35.09	periplasm
bglu_1g11450	phosphate ABC transporter periplasmic protein	128.26	38.48	inner
bglu_1g16080	family 1 extracellular solute-binding protein	175.94	46.70	periplasm
bglu_1g17730	Lipocalin family protein	6.07	6.49	outer
bglu_1g18060	ABC amino acid transporter, periplasmic ligand binding protein	27.29	25.53	periplasm
bglu_1g19550	Extracellular solute-binding protein, family 3	57.60	46.97	inner
bglu_1g22010	Sugar ABC transporter, periplasmic sugar-binding protein	42.60	23.18	inner
bglu_1g25530	Probably involved in high-affinity Fe2 transport	8.80	20.22	inner
bglu_1g29490	ABC sugar transporter, periplasmic ligand binding protein	10.90	11.31	periplasm
bglu_1g29540	Putative cation ABC transporter, periplasmic cation-binding protein	4.51	5.38	inner

bglu_1g29610	Transporter, hydrophobe/amphiphile efflux-1 (HAE1) family	6.54	1.98	inner
bglu_1g29620	Efflux transporter, RND family, MFP subunit	164.23	39.44	outer
bglu_1g31490	Phosphotransferase system, IIbc component	2.26	3.71	inner
bglu_1g32350	Peptide ABC transporter, periplasmic peptide-binding protein	83.99	21.97	periplasm
bglu_1g33350	ABC oligopeptide transporter, periplasmic ligand binding protein	238.80	34.57	inner
bglu_2g04040	Extracellular solute-binding protein, family 1	4.65	5.81	periplasm
bglu_2g04800	Glycine betaine/L-proline ABC transporter, periplasmic glycine betaine/L-proline-binding protein	10.02	13.61	periplasm
bglu_2g05910	proline/glycine betaine ABC transporter periplasmic protein	69.70	41.11	periplasm
bglu_2g09090	lipocalin family protein	24.20	17.73	outer
bglu_2g10360	Extracellular solute-binding protein, family 5	7.55	6.06	inner
bglu_2g13910	ABC-type dipeptide transport system, periplasmic component	4.46	4.73	periplasm

bglu_2g14790	Iron chelate uptake ABC transporter, FeCT family, ATP-binding protein	2.63	6.13	cytoplasm
bglu_2g14920	Electron transport protein SCO1/SenC	14.08	12.62	inner
bglu_2g20040	MFS efflux pump, membrane fusion protein, EmrA subfamily	2.04	5.84	inner
bglu_2g21400	Cationic amino acid ABC transporter, periplasmic binding protein	311.06	55.77	periplasm
**Membrane protein, Secretion system**				
bglu_1g00340	General secretion pathway protein G	14.46	14.67	inner
bglu_1g00380	General secretory pathway protein D	7.54	7.45	outer
bglu_1g01710	FliC	3.51	4.17	inner
bglu_1g03870	Type VI secretion system protein TssJ	118.27	30.73	outer
bglu_1g03880	TPR repeat-containing protein, type VI secretion system protein	389.34	60.28	outer
bglu_1g03910	Type VI secretion system protein TssD	28.43	40.12	cytoplasm
bglu_1g06040	Protein export protein SecF	3.20	5.38	inner
bglu_1g06050	Protein export protein SecD	8.45	5.17	inner

bglu_1g06060	Preprotein translocase, YajC subunit	2.32	18.52	inner
bglu_1g19750	Flp pilus assembly protein TadD contains TPRrepeats	2.21	4.93	outer
bglu_1g23930	Type IV secretory pathway VirD4 components-like protein	2.27	1.28	inner
bglu_1g31290	TPR domain protein	169.05	35.67	inner
bglu_1g32900	Secretion protein HlyD	13.73	4.96	inner
bglu_2g06400	Type VI secretion system protein	7.08	7.35	cytoplasm
bglu_2g10220	Type I secretion outer membrane protein	53.31	23.20	outer
bglu_2g10230	Twin-arginine translocation pathway signal	2.84	1.60	outer
bglu_2g12420	Type V secretory pathway, adhesin AidA	2.18	2.90	outer
**Membrane protein, Signal transduction**				
bglu_1g02980	Penicillin-binding protein, 1A family	23.60	10.46	inner
bglu_1g13280	Membrane carboxypeptidase/penicillin-binding protein	35.68	14.93	inner
bglu_1g14220	Transcriptional regulator, BadM/Rrf2 family	6.99	11.54	inner

bglu_1g18680	Periplasmic binding protein/LacI transcriptional regulator	159.02	48.43	inner
bglu_1g19950	GTP-binding protein TypA	4.93	3.14	inner
bglu_1g28210	Putative TonB-dependent vitamin B12 receptor BtuB	26.17	18.63	inner
bglu_1g32250	Transcriptional regulator, IclR family	2.09	4.50	inner
bglu_1g32870	LysR family transcriptional regulator	5.03	8.14	inner
bglu_1g34260	Transcriptional regulator, LysR family	5.63	8.95	inner
bglu_1p0980	Methyl-accepting chemotaxis sensory transducer	2.35	4.47	inner
bglu_2g04020	Transcriptional regulator, LacI family	18.33	4.02	inner
bglu_2g04350	Periplasmic binding protein/LacI transcriptional regulator	96.61	40.89	periplasm
bglu_2g09800	Outer membrane receptor for ferric coprogen and ferric-rhodotorulic acid	225.94	41.99	cytoplasm
bglu_2g11240	TonB-dependent siderophore receptor	5.01	4.11	cytoplasm
bglu_2g12250	TonB-dependent siderophore receptor	10.27	5.60	inner

bglu_2g12410	Penicillin-binding protein 1C	30.97	11.70	inner
bglu_2g12480	Multi-sensor signal transduction histidine kinase	22.31	1.95	inner
bglu_2g13410	Extracellular ligand-binding receptor	6.40	5.47	periplasm
bglu_2g14960	TonB-dependent receptor	45.77	18.67	outer
bglu_2g14990	TonB-dependent siderophore receptor	67.10	26.95	outer
bglu_2g16600	TonB-dependent receptor	7.70	4.28	outer
bglu_2g19860	Putative methyl-accepting chemotaxis protein	2.02	4.71	inner

Membrane protein, Enzyme

bglu_1g01590	Acid phosphatase AcpA	116.21	28.35	inner
bglu_1g02500	DNA-directed RNA polymerase, beta subunit	5.27	1.17	inner
bglu_1g04310	Thiol-disulfide interchange protein DsbC	3.45	7.47	periplasm
bglu_1g04510	AMP-dependent synthetase and ligase	2.27	2.69	inner
bglu_1g06110	Ferritin, Dps family protein	9.45	24.07	cytoplasm
bglu_1g06490	Mannose-1-phosphateguanylyltransferase/m	4.56	3.58	inner

	annose- 6-phosphate isomerase			
bglu_1g06660	UDP-3-0-acyl N-acetylglucosamine deacetylase	2.00	3.26	inner
bglu_1g08000	Pyrimidine reductase, riboflavin biosynthesis	2.18	3.23	inner
bglu_1g08380	Chorismate mutase	2.43	5.61	periplasm
bglu_1g08500	Disulfide bond formation protein DsbB	2.59	13.69	inner
bglu_1g09280	CreA protein	9.55	18.35	periplasm
bglu_1g09400	Ribonuclease E	4.86	2.31	inner
bglu_1g09590	Sigma factor algU regulatory protein MucB	13.00	8.57	periplasm
bglu_1g09640	Ribonuclease III	2.22	3.98	inner
bglu_1g10430	Thiamine biosynthesis protein ThiC	2.31	2.33	inner
bglu_1g11370	Carbamoyl-phosphate synthase, large subunit	3.29	1.66	inner
bglu_1g12790	Predicted membrane-associated Zn-dependent proteases	6.31	3.04	inner
bglu_1g12980	Inosine-5'-monophosphate dehydrogenase	2.57	4.12	inner
bglu_1g13560	Peptidyl-prolyl cis-trans isomerase D	6.80	3.55	inner

bglu_1g13650	PpiC-type peptidyl-prolyl cis-trans isomerase	25.10	11.97	periplasm
bglu_1g14090	Glycosyl transferase, group 2 family protein	2.22	2.96	inner
bglu_1g14120	Glycosyl transferase, family 39	2.15	2.13	inner
bglu_1g19570	Oxidoreductase, Gfo/Idh/MocA family	2.54	9.91	inner
bglu_1g22230	Predicted acyl-CoA transferases/carnitine dehydratase	2.01	2.96	inner
bglu_1g25980	Polyribonucleotide nucleotidyltransferase	34.29	8.46	inner
bglu_1g26640	3-carboxymuconate cyclase-like protein	11.09	8.98	periplasm
bglu_1g26720	Cytochrome c553	8.04	14.29	periplasm
bglu_1g27120	Pyocin R2_PP, tail length determination protein	2.00	1.93	inner
bglu_1g28000	Superoxide dismutase, Cu-Zn	33.92	26.29	outer
bglu_1g29070	Superoxide dismutase	2.13	4.17	periplasm
bglu_1g29130	3-hydroxyacyl-CoA dehydrogenase	2.10	7.54	periplasm
bglu_1g29270	N-acetylmuramoyl-L-alanine amidase	6.98	8.12	inner

bglu_1g30610	Parvulin-like peptidyl-prolyl isomerase	176.60	40.58	periplasm
bglu_1g31980	3-hydroxyacyl-CoA dehydrogenase, NAD-binding	7.49	3.53	inner
bglu_1g32090	D-alanyl-D-alanine carboxypeptidase family protein	165.44	54.04	inner
bglu_1g32210	Patatin family phospholipase	6.55	3.07	inner
bglu_1g32330	Thiol-disulfide interchange protein DsbA	30.14	17.45	periplasm
bglu_1g32390	Acetyl-CoA acetyltransferase	4.27	4.03	inner
bglu_1g33240	Putative transglycosylase	43.62	27.54	outer
bglu_2g03150	Putative carboxypeptidase-related protein	5.48	2.80	periplasm
bglu_2g03200	Aerobic-type carbon monoxide dehydrogenase	2.27	1.34	inner
bglu_2g05010	Sarcosine oxidase, alpha subunit family	2.02	1.50	inner
bglu_2g07230	Peptidylprolyl isomerase, FKBP-type	8.39	25.00	periplasm
bglu_2g08210	Malate/lactate dehydrogenases	8.44	10.40	inner
bglu_2g08230	Succinate dehydrogenase, cytochrome b subunit	2.43	7.25	inner
bglu_2g08250	Succinate dehydrogenase,	71.86	34.18	inner

	flavoproteinsubunit			
bglu_2g08280	Citrate synthase I	2.45	4.15	inner
bglu_2g08800	Fimbrial biogenesis outer membrane usher protein	11.61	1.48	outer
bglu_2g13350	Omega-amino acid--pyruvate aminotransferase	21.10	5.38	inner
bglu_2g14000	Class A beta-lactamase	18.96	16.89	outer
bglu_2g14940	Copper resistance protein CopC	6.79	25.41	outer
bglu_2g15990	dienelactone hydrolase family protein	13.88	18.21	inner
bglu_2g17110	Ubiquinol oxidase, subunit I	2.42	1.79	inner
bglu_2g17120	Ubiquinol oxidase polypeptide II precursor	2.83	5.07	inner
bglu_2g17310	Aminotransferase, class I and II	4.94	11.19	inner
bglu_2g18010	Neutral trehalase	2.22	2.22	outer
bglu_2g19560	Trans-sulfuration enzyme family protein	5.17	7.21	inner
bglu_2g22720	Squalene-hopene cyclase	13.26	7.47	inner
bglu_2p0610	Beta-ketoacyl synthase	2.69	1.51	inner
**Membrane protein, Lipoprotein**				
bglu_1g01050	Putative lipoprotein	3.57	16.42	outer
bglu_1g01920	Putative lipoprotein	10.57	14.82	outer

bglu_1g02250	Lipoprotein	23.28	10.77	outer
bglu_1g03230	VacJ-like lipoprotein	27.36	21.04	outer
bglu_1g04870	Lipoprotein	136.45	39.20	inner
bglu_1g05010	Outer membrane lipoprotein	586.40	51.27	outer
bglu_1g05420	Outer membrane lipoprotein, SmpA/OmlA	134.82	35.67	outer
bglu_1g05470	Lipoprotein, RlpB family	92.77	49.73	outer
bglu_1g08140	Outer membrane lipoprotein carrier protein LolA	25.59	21.03	inner
bglu_1g08250	Putative lipoprotein	34.51	44.85	periplasm
bglu_1g08450	Putative lipoprotein	49.83	33.05	outer
bglu_1g11910	Lipoprotein NlpD	29.02	33.05	outer
bglu_1g12190	RND efflux system, outer membrane lipoprotein, NodT family	2.17	2.46	outer
bglu_1g13870	Lipoprotein	1110.15	54.87	outer
bglu_1g14650	Lipoprotein	4.93	14.69	inner
bglu_1g14680	Competence lipoprotein ComL	61.49	27.76	inner
bglu_1g20160	Lipoprotein	729.64	61.50	outer
bglu_1g21930	Putative lipoprotein	27.94	23.53	outer
bglu_1g24380	NlpBDapX family lipoprotein	191.05	50.66	outer

bglu_1g27690	Lipoprotein	15.66	28.44	outer
bglu_1g29600	RND efflux system, outer membrane lipoprotein, NodT	244.79	31.68	outer
bglu_1g29890	Putative lipoprotein	7.07	9.17	outer
bglu_1g31300	Outer membrane lipoprotein LolB	46.31	41.12	outer
bglu_1g31570	Lipoprotein, YaeC family	67.87	31.73	periplasm
bglu_2g05350	Putative lipoprotein transmembrane	13.39	14.19	outer
bglu_2g07710	Lipoprotein NlpD	249.44	59.85	cytoplasm
bglu_2g09380	Putative lipoprotein transmembrane	27.19	10.40	outer
bglu_2g11680	Putative lipoprotein	4.63	11.48	outer
bglu_2g17660	Osmotically-inducible lipoprotein OsmE	2.34	10.17	periplasm
**Periplasmic protein, Enzyme**				
bglu_1g02420	Lipase/acylhydrolase	5.61	9.09	periplasm
bglu_1g03240	Toluene tolerance protein	129.99	38.94	periplasm
bglu_1g06030	YceI-like family protein	170.95	36.46	periplasm
bglu_1g09600	Do family protease	279.65	40.04	periplasm
bglu_1g19880	Putative exported heme utilisation related protein	6.47	3.75	outer

bglu_1g23640	Peptidyl-prolyl cis-trans isomerase A	27.21	21.35	periplasm
bglu_1g27570	Ecotin	92.24	22.35	periplasm
bglu_1g29660	Protease Do	1174.82	51.61	periplasm
bglu_1g29710	Phospholipase/lecithinase/hemolysin-like protein	8.19	10.53	periplasm
bglu_1g31800	Periplasmic protease	520.77	45.95	periplasm
bglu_2g02250	Probable awr type III effector family protein	49.95	2.86	cytoplasm
bglu_2g03500	Purine nucleoside permease	13.00	11.58	outer
bglu_2g11590	Inosine/uridine-preferring nucleoside hydrolase	69.55	31.69	periplasm
bglu_2g12650	Pyrrolo-quinoline quinone	2.42	3.70	inner
bglu_2g13610	Transglutaminase, N-terminal domain protein	2.04	0.95	cytoplasm
bglu_2g13670	Alkyl hydroperoxide reductase/ Thiol specific antioxidant/ Mal allergen	43.64	31.55	cytoplasm
bglu_2g22650	Toluene tolerance family protein	49.02	35.35	periplasm
bglu_2p0620	Putative exported avidin family protein	14.70	10.46	outer
**Membrane protein, Unclassified**				
bglu_1g02430	Translation elongation factor Tu	63.67	27.27	inner

bglu_1g04450	MltA domain protein	29.50	25.37	inner
bglu_1g00860	Hypothetical protein	60.68	38.52	periplasm
bglu_1g01000	Hypothetical protein	29.34	18.28	inner
bglu_1g01110	Hypothetical protein	2.32	9.79	periplasm
bglu_1g02190	Hypothetical protein	19.83	10.22	outer
bglu_1g02930	Hypothetical protein	2.04	2.73	inner
bglu_1g03990	Hypothetical protein	87.97	16.27	inner
bglu_1g05970	Hypothetical protein	29.79	29.88	inner
bglu_1g06870	Hypothetical protein	6.00	24.00	inner
bglu_1g08740	Hypothetical protein	69.82	32.50	periplasm
bglu_1g08890	Hypothetical protein	79.48	32.86	inner
bglu_1g09810	SEFIR domain-containing protein	2.35	8.73	inner
bglu_1g11100	Hypothetical protein	44.23	9.69	outer
bglu_1g11330	Hypothetical protein	13.97	5.23	outer
bglu_1g12320	Patatin	165.32	44.26	outer
bglu_1g12820	Hypothetical protein	12.67	19.05	periplasm
bglu_1g12990	Hypothetical protein	6.05	13.55	inner
bglu_1g14410	Peptidoglycan-binding LysM	154.90	35.76	inner
bglu_1g14530	Hypothetical protein	58.67	14.44	outer
bglu_1g15240	Hypothetical protein	5.07	11.45	outer

bglu_1g16360	Hypothetical protein	44.55	32.04	periplasm
bglu_1g18590	Predicted membrane protein	4.65	6.46	inner
bglu_1g19500	Hypothetical protein	4.72	8.22	inner
bglu_1g21950	Hypothetical protein	13.11	12.96	periplasm
bglu_1g23630	Tetratricopeptide repeat protein	6.22	7.56	periplasm
bglu_1g24190	YeeP	2.45	5.94	inner
bglu_1g24210	Hypothetical protein	2.23	5.22	outer
bglu_1g24490	Hypothetical protein	16.95	16.03	inner
bglu_1g25310	Rhs family protein	21.29	1.63	inner
bglu_1g25640	Hypothetical protein	6.89	12.08	periplasm
bglu_1g26000	Hypothetical protein	43.35	27.11	outer
bglu_1g26220	Hypothetical protein	4.60	16.59	inner
bglu_1g26450	Hypothetical protein	274.96	46.46	inner
bglu_1g27760	Hypothetical protein	60.76	37.50	periplasm
bglu_1g28050	Surface antigen (D15)	2.17	2.45	inner
bglu_1g28250	Hypothetical protein	163.21	42.62	periplasm
bglu_1g29650	Hypothetical protein	67.04	25.35	outer
bglu_1g30620	Hypothetical protein	83.41	22.68	inner
bglu_1g30980	Hypothetical protein	13.21	11.81	outer
bglu_1g31440	Hypothetical protein	13.74	22.22	outer

bglu_1g31550	Hypothetical protein	6.83	4.08	inner
bglu_1g32420	Hypothetical protein	2.13	2.66	periplasm
bglu_1g34050	Hypothetical protein	55.09	48.47	outer
bglu_2g01870	Hypothetical protein	2.69	3.81	inner
bglu_2g05460	Hypothetical protein	13.35	5.72	outer
bglu_2g06020	Hypothetical protein	9.73	15.17	outer
bglu_2g08190	Hypothetical protein	151.08	34.10	periplasm
bglu_2g09060	Hypothetical protein	2.56	2.90	inner
bglu_2g09660	Hypothetical protein	2.70	23.58	periplasm
bglu_2g10910	Hypothetical protein	57.59	42.16	outer
bglu_2g11750	Hypothetical protein	20.05	14.71	outer
bglu_2g12370	Hypothetical protein	5.94	9.40	periplasm
bglu_2g12400	Alpha-2-macroglobulin-like protein	106.74	17.09	periplasm
bglu_2g13920	Hypothetical protein	4.28	6.15	inner
bglu_2g14150	MltA-interacting MipA	2.36	6.39	outer
bglu_2g14930	Hypothetical protein	31.28	27.03	periplasm
bglu_2g16560	Hypothetical protein	4.48	16.54	inner
bglu_2g16570	Hypothetical protein	2088.24	72.31	outer
bglu_2g17300	Hypothetical protein	8.38	24.06	periplasm
bglu_2g17860	CsgG family protein	17.92	25.88	outer

bglu_2g19460	Hypothetical protein	34.52	38.79	periplasm
bglu_2g19820	Sel1 repeat-containing protein	2.31	3.59	outer
bglu_2g19830	Hypothetical protein	6.90	7.64	outer
bglu_3p0470	Rhs family protein	2.20	1.16	inner
bglu_3p0650	Hypothetical protein	6.46	11.01	inner
bglu_3p0980	Hypothetical protein	2.05	2.29	inner
bglu_4p0120	Hypothetical protein	2.09	5.31	inner
bglu_4p0300	Hypothetical protein	2.45	4.69	inner
bglu_4p1000	Hypothetical protein	8.85	5.45	outer
**Cytoplasmic, Protein folding**				
bglu_1g06310	Putative heat shock protein	2.00	12.59	cytoplasm
bglu_1g06340	Chaperone protein DnaK	15.80	4.29	cytoplasm
bglu_1g07140	Chaperonin Cpn10	3.60	27.84	cytoplasm
bglu_1g07150	Chaperonin GroEL	58.17	19.05	cytoplasm
bglu_1g26820	Molecular chaperone, HSP90 family	2.47	2.22	cytoplasm
**Cytoplasmic, Regulatory protein**				
bglu_1g02550	Elongation factor EF-2	24.32	9.43	cytoplasm
bglu_1g02840	DNA-directed RNA polymerase, alpha subunit	2.43	7.08	cytoplasm
bglu_1g04960	Ribonucleotide-diphosphate	2.08	1.50	cytoplasm

	reductase alpha subunit			
bglu_1g06100	Oxidative stress regulatory protein OxyR	5.42	6.27	cytoplasm
bglu_1g10680	Acyl-homoserine lactone dependent transcriptional activator	2.08	6.22	cytoplasm
bglu_1g16630	AMP-dependent synthetase and ligase	4.22	5.20	cytoplasm
bglu_1g20020	Translation initiation factor IF-2	2.00	0.82	cytoplasm
bglu_1g21480	Phenylalanyl-tRNA synthetase, alpha subunit	4.93	10.98	cytoplasm
bglu_1g28770	Isoleucyl-tRNA synthetase	8.82	1.90	cytoplasm
bglu_1g29960	Regulatory protein RecX	2.40	9.70	cytoplasm
bglu_1g32600	YbaK/prolyl-tRNA synthetase associated region	11.41	12.87	cytoplasm
bglu_2g01310	GntR domain protein	6.39	9.88	cytoplasm
bglu_2g02050	Transcriptional regulator	2.14	7.65	cytoplasm
bglu_2g02260	Transcriptional regulator, winged helix family	40.38	4.42	cytoplasm
bglu_2g06540	Transcriptional regulator, MarR family	2.19	11.46	cytoplasm
bglu_2g08590	DNA-binding protein BpH	15.11	29.25	cytoplasm
bglu_2g12270	Sigma-24 (FecI-like) protein	7.44	17.75	cytoplasm

bglu_2g19020	Transcriptional regulator, MocR family	4.49	4.66	cytoplasm
bglu_2g19400	Autoinducer-binding domain protein	2.00	6.59	cytoplasm
**Cytoplasmic, Enzyme**				
bglu_1g01670	DegT/DnrJ/EryC1/StrS aminotransferase	2.89	7.07	cytoplasm
bglu_1g01990	S-adenosyl-L-homocysteine hydrolase	6.13	3.60	cytoplasm
bglu_1g04490	NAD-dependent aldehyde dehydrogenases	2.46	2.84	cytoplasm
bglu_1g06400	Poly(A) polymerase	2.14	2.86	cytoplasm
bglu_1g07350	Glycosyl transferase	7.20	4.56	cytoplasm
bglu_1g08440	Peptidase M1, alanyl aminopeptidase	4.15	1.11	cytoplasm
bglu_1g10580	Pyruvate dehydrogenase E1 component	2.54	2.70	cytoplasm
bglu_1g11920	Aldose 1-epimerase	2.04	7.28	cytoplasm
bglu_1g12350	Anaerobic dehydrogenase	2.09	1.45	cytoplasm
bglu_1g12850	UDP-N-acetylglucosamine acyltransferase	2.16	4.96	cytoplasm
bglu_1g12900	Phosphoenolpyruvate synthase	2.12	1.63	cytoplasm
bglu_1g13630	Dihydroxy-acid dehydratase	4.20	4.36	cytoplasm

bglu_1g24510	Enolase	2.59	4.22	cytoplasm
bglu_1g25910	NADH dehydrogenase delta subunit	2.22	2.64	cytoplasm
bglu_1g27940	Phosphoenolpyruvate carboxylase	2.19	0.97	cytoplasm
bglu_1g28550	Pseudouridine synthase, Rsu	2.04	7.11	cytoplasm
bglu_1g28910	Isocitrate dehydrogenase, NADP-dependent	10.22	7.55	cytoplasm
bglu_1g29320	Pyridoxamine 5'-phosphate oxidase	4.39	13.89	cytoplasm
bglu_1g29930	Succinyl-CoA synthase, alpha subunit	2.93	5.80	cytoplasm
bglu_1g33680	S-adenosylmethionine synthetase	2.87	4.55	cytoplasm
bglu_1g33940	Putative rod shape-determining protein	2.39	8.93	cytoplasm
bglu_2g02870	Squalene-hopene cyclase	5.28	3.99	cytoplasm
bglu_2g03230	Serine hydroxymethyltransferase 2 (Serine methylase 2)	6.47	10.00	cytoplasm
bglu_2g03530	Alpha/beta hydrolase	160.77	5.65	cytoplasm
bglu_2g08260	Succinate dehydrogenase and fumarate reductase iron-sulfur protein	6.90	5.58	cytoplasm

bglu_2g10840	Possible LysM domain	2.57	4.15	cytoplasm
bglu_2g11560	Alkylhydroperoxidase like protein, AhpD family	16.93	9.72	cytoplasm
bglu_2g11930	Acyl-CoA dehydrogenases	3.04	8.00	cytoplasm
bglu_2g17390	Nitrate reductase, alpha subunit	7.30	1.66	cytoplasm
bglu_2g22300	Pyrroloquinoline quinone biosynthesis protein PqqE	2.65	9.16	cytoplasm
bglu_2p0500	Integrase, catalytic region	11.18	6.40	cytoplasm
**Cytoplasmic, Ribosomal protein**				
bglu_1g02470	Ribosomal protein L1	6.21	10.34	cytoplasm
bglu_1g02540	30S ribosomal protein S7	3.11	10.26	cytoplasm
bglu_1g02700	50S ribosomal protein L5	10.92	13.97	cytoplasm
bglu_1g02760	50S ribosomal protein L30	5.10	23.81	cytoplasm
bglu_1g02850	50S ribosomal protein L17	7.03	13.85	cytoplasm
bglu_1g05690	Ribosomal protein L13	10.57	17.61	cytoplasm
bglu_1g08800	Ribosomal protein S1	23.07	12.15	cytoplasm
bglu_1g09030	30S ribosomal protein S16	5.61	32.14	cytoplasm
bglu_1g13970	50S ribosomal protein L9	6.26	8.00	cytoplasm
**Cytoplasmic, Hypothetical**				
bglu_1g12300	Hypothetical protein	4.79	2.91	cytoplasm
bglu_1g17140	Hypothetical protein	7.64	5.51	cytoplasm

bglu_1g27660	Hypothetical protein	2.22	6.98	cytoplasm
bglu_2g07480	Hypothetical protein	2.04	2.48	cytoplasm
bglu_2g10940	Hypothetical protein	2.05	2.84	cytoplasm
bglu_2g17990	Hypothetical protein	4.24	17.36	cytoplasm
bglu_2g18790	Hypothetical protein	2.23	3.15	cytoplasm
bglu_4p1010	Hypothetical protein	4.59	2.85	cytoplasm
**Unclassified, DNA recombination**				
bglu_1g00410	DNA-binding protein HU-alpha	8.33	36.96	cytoplasm
bglu_1g18980	DNA replication protein	2.43	7.29	inner
bglu_1g19080	Nucleoside diphosphate kinase	2.45	13.18	inner
bglu_1g19130	Nucleoside diphosphate kinase	2.82	8.01	cytoplasm
bglu_1g21880	MTA/SAH nucleosidase	5.28	12.69	inner
bglu_1g24320	Putative integrase	2.46	3.70	cytoplasm
bglu_1g25540	Excinuclease ABC, B subunit	2.54	2.73	cytoplasm
bglu_1g29240	Site-specific tyrosine recombinase XerD	5.11	5.87	cytoplasm
bglu_1g32320	Cell division protein	6.17	4.48	inner
bglu_1p0510	Phage integrase family protein	2.04	2.73	inner
bglu_2g07270	Excinuclease ABC, A subunit	2.10	1.02	inner
bglu_3p0520	TrwC protein	4.17	1.34	cytoplasm
bglu_4p0020	Chromosome partitioning	2.04	4.41	cytoplasm

	protein ParB			
bglu_4p1120	Phage integrase family protein	4.70	2.63	cytoplasm

a: *B. glumae* BGR1 genome database; GenBank accession number: CP001503–CP001508.

b: Raw MS/MS data were processed with Proteome Discoverer™. Peptides with at least six amino acids were considered for identification. The false discovery rates for both peptides and proteins were set to 0.01.

c: Subcellular localization of each vesicular protein was predicted based on PSORTb version 3.02 (www.psort.org/psortb/). Inner and outer denote the inner and outer membranes, respectively.

References

Simon, R., Priefer, U. & Pühler, A. A broad host range mobilization system for *in vivo* genetic engineering: transposon mutagenesis in Gram negative bacteria. *Bio/Technology* **1**, 784–791 (1983).

Kim, J. *et al.* Quorum sensing and the LysR-type transcriptional activator ToxR regulate toxoflavin biosynthesis and transport in *Burkholderia glumae*. *Mol Microbiol* **54**, 921–934 (2004).

Kim, J. *et al.* Regulation of polar flagellum genes is mediated by quorum sensing and FlhDC in *Burkholderia glumae*. *Mol Microbiol* **64**,165–179 (2007).

Staskawicz, B., Dahlbeck, D., Keen, N. & Napoli, C. Molecular characterization of cloned avirulence genes from race 0 and race 1 of *Pseudomonas syringae* pv. *glycinea*. *J Bacteriol* **169**, 5789–5794 (1987).
